# A 3-way hybrid approach to generate a new high-quality chimpanzee reference genome (Pan_tro_3.0)

**DOI:** 10.1093/gigascience/gix098

**Published:** 2017-10-30

**Authors:** Lukas F K Kuderna, Chad Tomlinson, LaDeana W Hillier, Annabel Tran, Ian T Fiddes, Joel Armstrong, Hafid Laayouni, David Gordon, John Huddleston, Raquel Garcia Perez, Inna Povolotskaya, Aitor Serres Armero, Jèssica Gómez Garrido, Daniel Ho, Paolo Ribeca, Tyler Alioto, Richard E Green, Benedict Paten, Arcadi Navarro, Jaume Betranpetit, Javier Herrero, Evan E Eichler, Andrew J Sharp, Lars Feuk, Wesley C Warren, Tomas Marques-Bonet

**Affiliations:** 1 Institut de Biologia Evolutiva, (CSIC-Universitat Pompeu Fabra), PRBB, Doctor Aiguader 88, Barcelona, Catalonia 08003, Spain; 2 CNAG-CRG, Centre for Genomic Regulation (CRG), Barcelona Institute of Science and Technology (BIST), Baldiri i Reixac 4, 08028, Barcelona, Spain; 3 McDonnell Genome Institute, Department of Medicine, Department of Genetics, Washington University School of Medicine, 4444 Forest Park Ave., St. Louis, MO 63108, USA; 4 Bill Lyons Informatics Centre, UCL Cancer Institute, University College London, 72 Huntley Street, London WC1E 6DD, UK; 5 Genomics Institute, University of California Santa Cruz and Howard Hughes Medical Institute, 1156 High Street, Santa Cruz, CA 95064, USA; 6 Bioinformatics Studies, ESCI-UPF, Pg. Pujades 1, 08003, Barcelona, Spain; 7 Department of Genome Sciences, University of Washington School of Medicine, Box 355065, Seattle, WA 98195, USA; 8 Howard Hughes Medical Institute, University of Washington, Box 355065, Seattle, WA 98195, USA; 9 Department of Genetics and Genomic Sciences, Icahn School of Medicine at Mount Sinai, New York, NY 10029, USA; 10 The Pirbright Institute, Ash Road, Pirbright, Woking, GU24 0NF, UK; 11 Department of Biomolecular Engineering, University of California Santa Cruz, 1156 High Street, Santa Cruz, CA 95060, USA; 12 Dovetail Genomics, Santa Cruz, 2161 Delaware Ave., Santa Cruz, CA 95060, USA; 13 Institucio Catalana de Recerca i Estudis Avancats (ICREA), Passeig Lluís Companys 23, Barcelona, Catalonia 08010, Spain; 14 Department of Immunology, Genetics and Pathology, Science for Life Laboratory, Box 815, Uppsala University 751 08 Uppsala, Sweden

**Keywords:** chimpanzee reference genome, assembly, genomics

## Abstract

The chimpanzee is arguably the most important species for the study of human origins. A key resource for these studies is a high-quality reference genome assembly; however, as with most mammalian genomes, the current iteration of the chimpanzee reference genome assembly is highly fragmented. In the current iteration of the chimpanzee reference genome assembly (Pan_tro_2.1.4), the sequence is scattered across more then 183 000 contigs, incorporating more than 159 000 gaps, with a genome-wide contig N50 of 51 Kbp. In this work, we produce an extensive and diverse array of sequencing datasets to rapidly assemble a new chimpanzee reference that surpasses previous iterations in bases represented and organized in large scaffolds. To this end, we show substantial improvements over the current release of the chimpanzee genome (Pan_tro_2.1.4) by several metrics, such as increased contiguity by >750% and 300% on contigs and scaffolds, respectively, and closure of 77% of gaps in the Pan_tro_2.1.4 assembly gaps spanning >850 Kbp of the novel coding sequence based on RNASeq data. We further report more than 2700 genes that had putatively erroneous frame-shift predictions to human in Pan_tro_2.1.4 and show a substantial increase in the annotation of repetitive elements. We apply a simple 3-way hybrid approach to considerably improve the reference genome assembly for the chimpanzee, providing a valuable resource for the study of human origins. Furthermore, we produce extensive sequencing datasets that are all derived from the same cell line, generating a broad non-human benchmark dataset.

## Data Description

### Creating a non-human sequencing benchmark dataset

To test the potentially combinatorial power of varied sequencing and mapping strategies, we created several different datasets on different platforms to try to leverage the advantages of each, as the shortcomings of 1 sequencing strategy might be compensated for by another [1]. All datasets are derived from a single male western chimpanzee (“Clint,” Coriell identifier S006007), the same individual used to generate the current Chimpanzee genome assembly. We produced ∼120-fold sequence coverage of overlapping 250-bp reads (∼450-bp fragment) on the Illumina HiSeq 2500 platform, offering high accuracy and throughput, but comparatively short reads; ∼9-fold sequence coverage from 43 Pacific Biosciences SMRT-Cells with P5-C3 chemistry on the RSII instrument, offering long reads at lower accuracy; Illumina TruSeq Synthetic long reads at around 2-fold coverage, offering long-range information derived from local assemblies of ∼10-Kb fragments [2]; 1 lane of *in vitro* proximity ligation read pairs (prepared as a Chicago library by Dovetail Genomics) [3] sequenced on the Illumina HiSeq 2000 platform, offering spatial contact information of the chromatin, that can be exploited for scaffolding.

These diverse datasets complement the resources that were already available for the same cell line, namely 6-fold coverage of ABI Sanger capillary reads used for the initial chimpanzee genome assembly, a 100-bp paired Illumina HiSeq data, a fosmid library at 6-fold physical coverage with available end sequences, a Bacterial Artificial Chromosome (BAC) library at 3-fold physical coverage with available end sequences and around 700 finished BACs [4]. Altogether, these data constitute an extensive non-human and non-model organism benchmarking dataset for different sequencing strategies.

### Assembly generation

We generated a complete *de novo* assembly for the chimpanzee with a combination of the datasets. At each step of our assembly, we measured increase in contiguity by means of the N50 statistic, which is defined as the length of a contig or scaffold such that 50% of the assembly bases are contained in contigs or scaffolds of at least that length. The starting point of our assembly scaffolding efforts are contigs generated with DISCOVAR *de novo* [5] from 250 bp of paired-end reads. These reads are derived from a 450-bp library, resulting in pairs that overlap over a ∼50-bp region, a feature that is exploited by the assembler. While based on Illumina sequencing, these libraries have recently been shown to produce assemblies superior in contiguity when compared to assemblies derived from conventional Illumina libraries [6]. The DISCOVAR base assembly had a contig N50 of 87 Kbp, and was then scaffolded using proximity ligation read-pairs generated by the Chicago method [3] and sequenced on the Illumina platform. These data increased the scaffold N50 to 26 Mbp. Notably, individual scaffolds exceed lengths of 75 Mbp, and therefore already reach the order of magnitude of full chromosomal arms. We sought to take advantage of these highly contiguous scaffolds and attempt closure of remaining gaps with long-read single-molecule sequences by PacBio using PBJelly (PBJelly, RRID:SCR_012091) [7]. By this means, we filled over 38 000 gaps (or 55%) among all scaffolds, and in so doing increased the contig N50 by over 320% to 283 Kbp when compared to the DISCOVAR base assembly (see Table 1). While we went on to further improve the assembly with additional data (see below), these statistics give an approximation of the contiguity that can be expected for *de novo* assemblies of previously unsequenced species using our 3-way hybrid approach: contigs derived from overlapping 250-bp paired-end reads to scaffold with *in vitro* HiC, and fill remaining gaps with PacBio data. When the contiguity metrics of this intermediate assembly are compared to other representative non-human primate genomes (as annotated by NCBI Refseq category, July 1, 2016; see the Supplementary Data), we observed superior contiguity in contig structure within our assembly compared to all others. The only exception is the gorilla genome, recently assembled from deep (∼75-fold) long-read sequences [8]. However, our stepwise method offers an approach that is considerably cheaper.

**Table 1:** Assembly statistics comparing the previous chimpanzee assembly, our intermediary assembly based on the 3-way hybrid and the finished assembly Pan_tro_3.0

	Pan_tro_2.1.4	3-way hybrid (intermediary)	Pan_tro_3.0
Scaffold N50, bp	8 925 874	26 681 610	26 972 556
Contig N50, bp	50 665	282 774	384 816
Contig N90, bp	7231	41 655	53 112
Assembly length, bp	3 309 577 923	2 992 696 208	3 231 154 112
Assembly length w/o Ns, bp	2 902 338 968	2 990 712 612	3 132 603 062
Scaffolds	24 129	45 000	44 448
Contigs	183 827	76 674	72 226
Gaps	159 698	31 674	26 715

In this context, we defined gaps at stretches of at least 10 consecutive “Ns” in the assembly. Contigs are defined as contiguous stretches of sequence without gaps.

### Assembly refinement and comparison to Pan_tro_2.1.4

For the final release of the chimpanzee assembly, we created a reference assembly that leveraged previous resources generated from the same individual [4]. First, we merged in regions from Pan_tro_2.1.4 that were derived from Clint and gapped in our assembly. It is known that Pan_tro_2.1.4 contains sequences from different chimpanzees. To do so, we extracted flanking sequence regions of gaps in our assembly and mapped all to Pan_tro_2.1.4, keeping only unique and concordant mappings that do not span any gaps within Pan_tro_2.1.4, and merged the spanned Pan_tro_2.1.4 sequence in.

To ensure that accuracy was not sacrificed for continuity gains, we utilized various methods to measure error. Given that our assembly likely contained some erroneous links between contigs or misassembled contigs as a result of *de novo* assembly, conformational mapping, or merging mistakes, we first used discordant mapping of fosmid end sequences (∼40-Kbp insert size) to identify any large misassemblies. We identified 17 such scaffold errors and manually broke apart each. We also sought to correct any remaining single base substitutions or small indels (<6 bp) with a series of custom mapping and base integration programs (see the Supplementary Data). With the same Illumina data used to generate the DISCOVAR base assembly, we corrected more than 500 000 single base or indel errors. Most of these residual errors are presumably derived from regions where PacBio data were incorporated into the assembly, as this platform is known to have an elevated error rate. As another measure of quality, we produced whole-genome alignments to Pan_tro_2.1.4 and found that our assembly aligns with, on average, 99.9% identity, and the magnitude of remaining differences can thus be reasonably explained by the allelic diversity of western chimpanzees [9].

**Figure 1:**
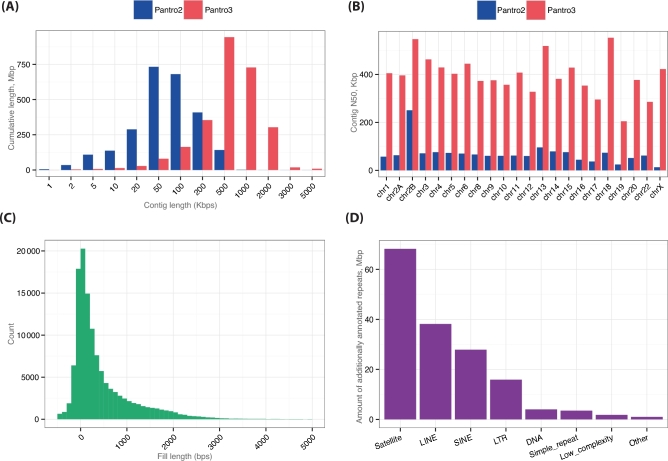
**(A)** Genome-wide distribution of contig lengths between Pan_tro_2.1.4 and Pan_tro_3.0. The peak for Pan_tro_3.0 is shifted to higher values by an order of magnitude. **(B)** Increase in contig N50 for all chromosomes that were not finished with clones in Pan_tro_2.1.4 or Pan_tro_3.0. **(C)** Length distribution of filled gaps in Pan_tro_3. Negative values constitute wrongly separated overlapping contig ends in Pan_tro_2.1.4. **(D)** Increase in annotated interspersed repeats separated by repeat family.

For our final assembly, named Pan_tro_3.0, we integrated previously available finished clone sequences derived from Clint where possible. Pan_tro_3.0 spans 2.95 Gbp in ordered and oriented chromosomal sequences. An additional 140 Mbp of sequence is assigned to chromosomes, but their order and orientation are unknown, and 123 Mbp remain of unknown chromosomal origin. Pan_tro_3.0 has a genome-wide contig and scaffold N50 of 385 Kbp and 27 Mbp, respectively, constituting an improvement in contiguity over Pan_tro_2.1.4 of 760% and 300%, respectively (see Fig. 1A and Table 1). We observed this increase across all non-finished chromosomes, with the most pronounced effect on the X chromosome (see Fig. 1B). This chromosome shows the highest degree of fragmentation in Pan_tro_2.1.4, likely due to the fact that the effective sequence coverage on the sex chromosomes is only half that of the autosomes, namely around 3-fold in the original assembly. We increased the contig N50 on the X chromosome by 3250% from 13 Kbp to 422 Kbp, thus bringing its contiguity to the range observed on autosomes.

Overall, we decreased the number of contigs by more than 60%, from 183 860 to 72 226, and the number of gaps by 83%, from 156 857 to 26 715. As gap structures between the assemblies may not correspond, we identified filled gaps from Pan_tro_2.1.4 by extracting their flanking regions and mapping them onto Pan_tro_3.0. By keeping only unique and concordant mappings that do not span any gaps in Pan_tro_3.0, we estimate the sequences of 122 943 (77%) gaps to be filled, amounting to 60.3 Mbp of sequence. The majority of these fill sequences are comparably short (see Fig. 1C) and significantly enriched in interspersed genomic repeats, with 58% of them (*P* < .0001, feature permutation test) intersecting with repeats. Of these, around 16 Mbp are fully embedded within fill sequences, corresponding to, amongst others, more than 29 650 novel short interspersed nuclear element (SINE) annotations and 20 888 novel long interspersed nuclear elements (LINE) annotations.

### Repeat resolution

Large genomic repeats constitute a major confounding factor in genome assembly and are therefore one of the main reasons for their fragmentation, and thus the assembly repeat representation can be a proxy of its quality. To assess the repeat resolution of interspersed repeats, we masked Pan_tro_3.0 using RepeatMasker (RepeatMasker, RRID:SCR_012954) [10], selecting chimpanzee-specific repeats, resulting in 1.64 Gbp (52.2%) being annotated as repeats. The proportion of repetitive elements is similar in Pan_tro_2.1.4 (50.9%); however, given the large amount of newly resolved sequences, this translates into a substantial increase in annotated repeats. Specifically, we annotate 164 Mbp of novel repeats in Pan_tro_3.0, comprising around 10% of the whole repeat annotation. We observe this increase consistently across all families of interspersed repeats (see Fig. 1D). The increases range as high as 300% for satellite sequences, corresponding to an additional 68.2 Mbp of newly resolved sequence in this category. We also increased the amount of annotated SINE by 27.9 Mbp, including 83 637 additional resolved copies of *Alu* elements. We find the increase in annotations to be negatively correlated with age for *Alu* elements, and thus find the highest increase (8.8%) for the youngest and least divergent subfamily (*AluY*), suggesting that common high-identity repeats are now better resolved. We furthermore added 38.2 Mbp of sequence annotated as LINEs to the assembly. We also observed a noteworthy increase in annotated long terminal repeats, adding 15.9 Mbp to this repeat category, corresponding to 30 574 additional annotations of endogenous retroviruses in the genome. When comparing all types of interspersed repeats between Pan_tro_2.1.4 and Pan_tro_3.0, we find a median increase of 4.7% of sequence, highlighting that repeat resolution is much improved in Pan_tro_3.0 (see Supplementray Table S4).

### Representation of segmental duplications

To analyze the representation of segmental duplications in Pan_tro_3.0, we applied 2 alternative approaches. First, we performed a whole-genome assembly comparison (WGAC) to compare repeat-free sequences of the assembly to itself [11]. This method identifies duplicated sequence in blocks of at least 1 Kbp with 90% identity or higher. Excluding unplaced contigs, we found 140 Mbp of non-redundant duplicated sequence in Pan_tro_3.0 chromosomes, or 4.46% of the non-gap bases in the assembly, results that are consistent with previous read-depth estimates for chimpanzee [12] and analyses of high-quality, finished human genome assemblies (see Supplementary Data S3). Second, we identified duplications by whole-genome shotgun sequence detection (WSSD), which identifies duplications at least 10 Kbp long with over 94% identity by detecting regions of increased read depth compared to known unique regions [13]. We used 31 366 275 Sanger capillary reads derived from Clint, and found 51 Mbp of duplicated sequence meeting these criteria on placed chromosomes, compared to 68 Mbp detected by WGAC.

Genome wide, we discovered 178 245 redundant pairwise alignments corresponding to 388 Mbp of non-redundant sequence greater than 1 Kbp in length and 90% identity (12.39% of the genome sequence excluding gaps) by WGAC, and 63 Mbp of duplicated sequence by WSSD (compared to 284 Mbp WGAC ≥10 Kbp, >94% identity). We then compared Pant_tro_3.0 to the human reference genome assembly GRCh38, an assembly that is based on a BAC hierarchical shotgun assembly strategy and may therefore be considered the gold standard with respect to representation of segmental duplications. We note similar proportions of bases in segmental duplications on chromosomal scaffolds (4.46% in Pan_tro_3.0 vs 5.56% in GRCh38); however, we note an elevated genome-wide rate of bases in duplications when including unplaced and unlocalized scaffolds. This suggests that our assembly includes false-positive paralogous regions (see Supplementary Table S1).

### Gene annotation

We produced a new gene annotation based on projections from all human transcripts in the GENCODE annotation V24 set combined with RNA-seq data derived from the brain, heart, liver, and testis from 3 different individuals [14]. To quantify the effect of the underlying sequence on the annotation, we annotated Pan_tro_2.1.4. with the same data. We observed improvements in gene annotation in Pan_tro_3.0 in all considered metrics. We increased the number of recovered consensus gene models for protein coding transcripts by 2.7% and are now able to project and annotate 89.5% of the GENCODE human coding transcripts onto the new assembly. The average coverage of these transcripts within the genome is 98.9%, a gain of 2%. We also observe an increase of 6.6% in transcripts with multiple mappings. We checked for newly resolved exonic sequences in filled gaps with respect to Pan_tro_2.1.4, and find 17 818 exons, amounting to 851 Kbp of non-overlapping sequence, to be fully embedded within them. Altogether, we retrieved models for 77 858 coding transcripts, corresponding to the isoforms of 20 373 coding genes.

We find 5039 human coding transcripts corresponding to 2660 genes with predicted frameshift mutations in Pan_tro_2.1.4 to human, but not in Pan_tro_3.0. Conversely, we find 674 genes with predicted frameshift mutations to human that are present in Pan_tro_3, but not in Pan_tro_2.1.4. Given that both assemblies are mainly based on data from the same individual (with the exception of chromosome 21 and around 28% of chromosome 7 in Pan_tro_2.1.4, which were derived from a different individual), the majority of these predictions constitute either allelic variation or putative sequence errors in Pan_tro_2.1.4.

In summary, we describe a hybrid assembly approach to obtain a more complete *de novo* chimpanzee reference genome assembly, substantially increasing contiguity metrics within it. Our proposed assembly method should be easily applicable to different organisms of similar genomic architecture.

## Availability of supporting data

We have corrected several orientation errors in the sequences described in this article. The corrected sequences can be found in the associated Gigascience Database.

Supporting data are available through the *Giga*DB database (*Giga*DB, RRID:SCR_004002) [15]. This whole-genome shotgun project has been deposited at DDBJ/ENA/GenBank under the accession AACZ00000000. The version described in this paper is version AACZ04000000. The assembly is available at https://www.ncbi.nlm.nih.gov/assembly/GCF_000001515.7 and at the UCSC genome browser under the identifier panTro5. The assembly denominated Pan_tro_2.1.4 in the manuscript refers to Pan_troglodytes-2.1.4 with the RefSeq assembly accession number GCF_000001515.6.

## Additional file

Kuderna_et_al.SUPPLEMENTARY_resubmission.docx

## Abbreviations

bp: base pairs; Kbp: kilo base pairs; Mbp: mega base pairs; indel: insertion-deletion; SINE: short interspersed nuclear element; LINE: long interspersed nuclear element; LTR: long terminal repeat; ERV: endogenous retrovirus; WGAC: whole-genome assembly comparison; WSSD: whole-genome shotgun sequence detection.

## Competing interests

EEE is on the Scientific Advisory Board (SAB) of DNAnexus, Inc. REG is the co-founder of Dovetail Genomics.

## Funding

J.G.G. is funded by the RED-BIO project of the Spanish National Bioinformatics Institute (INB) under grant number PT13/0001/0044. The INB is funded by the Spanish National Health Institute Carlos III (ISCIII) and the Spanish Ministry of Economy and Competitiveness (MINECO). L.F.K.K. is supported by an FPI fellowship associated with BFU2014-55090-P (FEDER); L.F. is supported by the Swedish Foundation for Strategic Research F06-0045 and the Swedish Research Council; E.E.E. is an investigator of the Howard Hughes Medical Institute. A.J.S. is supported by US National Institutes of Health (NIH) grants DA033660, HG006696, HD073731, and MH097018, and research grant 6-FY13-92 from the March of Dimes. This work was supported, in part, by grants from the NIH (grants R01HG002385 and U24HG009081 to E.E.E., HG007990 and HG007234 to B.P.). T.M.B. is supported by MINECO BFU2014-55090-P (FEDER), BFU2015-7116-ERC, and BFU2015-6215-ERC, Fundacio Zoo Barcelona and Secretaria d’Universitats i Recerca del Departament d’Economia i Coneixement de la Generalitat de Catalunya.

## Author contributions

T.M.B., W.C.W., and L.F. conceived the study. L.F.K.K., C.T., L.W.H., and R.E.G. produced and analyzed the assembly. I.F., J.A., J.G.G., T.A., B.P., A.T., H.L., J.B., R.G.P., I.P., A.S.A., J.He, P.R., D.H., A.N., and A.J.S. produced, analyzed, and interpreted the assembly and annotations. D.G., J.Hu, and E.E.E. analyzed segmental duplications. T.M.B., W.C.W., and L.F.K.K. wrote the manuscript with input from all authors.

## Supplementary Material

GIGA-D-16-00140_Original-Submission.pdf

GIGA-D-16-00140_Revision-1.pdf

Response-to-Reviewer-Comments_Original-Submission.pdf

Reviewer-1-Report-(Original-Submission).pdf

Reviewer-1-Report-(Revision-1).pdf

Reviewer-2-Report-(Original-Submission).pdf

Supplement materials
