# Supplementary material for: A 3-way hybrid approach to generate a new high-quality chimpanzee reference genome (Pan_tro_3.0)
Source: Gigascience. 2017 Oct 30;6(11):1–6. doi: 10.1093/gigascience/gix098 (PMC5714192; doi:10.1093/gigascience/gix098)
Supplement: GIGA-D-16-00140_Original-Submission.pdf [file gix098_giga-d-16-00140_original-submission.pdf]

GigaScience

A 3-way hybrid approach to generate a new high quality chimpanzee reference genome (Pan_tro_3.0)

--Manuscript Draft--

Manuscript Number:	GIGA-D-16-00140
Full Title:	A 3-way hybrid approach to generate a new high quality chimpanzee reference genome (Pan_tro_3.0)
Article Type:	Data Note

Funding Information:

Ministerio de Economía y Competitividad (BFU2014-55090-P)	Mr Tomas Marques-Bonet
Stiftelsen för Strategisk Forskning (F06-0045)	Mr Lars Feuk
National Institutes of Health (DA033660)	Mr Andrew J. Sharp
National Institutes of Health (HG006696)	Mr Andrew J. Sharp
National Institutes of Health (HD073731)	Mr Andrew J. Sharp
National Institutes of Health (MH097018)	Mr Andrew J. Sharp
March of Dimes Foundation (6-FY13-92)	Mr Andrew J. Sharp
Ministerio de Economía y Competitividad (BFU2015-7116-ERC)	Mr Tomas Marques-Bonet
Ministerio de Economía y Competitividad (BFU2015-6215-ERC)	Mr Tomas Marques-Bonet
National Institutes of Health (HG002385)	Mr Evan E. Eichler
National Institutes of Health (HG007990)	Mr Benedict Paten
National Institutes of Health (HG007234)	Mr Benedict Paten

Abstract:

Background:
The chimpanzee is arguably the most important species for the study of human origins. A key resource for these studies is a high quality reference genome assembly, however, as most mammalian genomes, the current iteration of the chimpanzee reference genome assembly it is highly fragmented. In the current iteration of the chimpanzees reference genome assembly (Pan_tro_2.1.4), the sequence is scattered across more then 183,000 contigs and incorporating over 159,000 gaps, with a genome wide contig N50 of 51 Kbp.

Findings:
In this work we produce an extensive and diverse array of sequencing datasets to rapidly assemble a new chimpanzee reference that surpasses previous iterations in bases represented and organized in large scaffolds. To this end, we show substantial improvements over the current release of the chimpanzee genome (Pan_tro_2.1.4) by several metrics, such as: increased contiguity by >750% and 300% on contigs and scaffolds, respectively; closure of 77% of gaps in the Pan_tro_2.1.4 assembly gaps spanning >850 Kbp of novel coding sequence based on RNASeq data. We furthermore report over 2,700 genes that had putatively erroneous frame-shift predictions to human in Pan_tro_2.1.4 and show a substantial increase in the annotation of repetitive elements.

Conclusions:
We apply a simple 3-way hybrid approach to considerably improve the reference genome assembly for the chimpanzee, providing a valuable resource to study human origins. We furthermore produced extensive sequencing datasets that are all derived from the same cell line, generating a broad non-human benchmark dataset.

Powered by Editorial Manager® and ProduXion Manager® from Aries Systems Corporation

**Corresponding Author:**	Lukas F. K. Kuderna<br><br>SPAIN
**Corresponding Author Secondary Information:**	
**Corresponding Author's Institution:**	
**Corresponding Author's Secondary Institution:**	
**First Author:**	Lukas F. K. Kuderna
**First Author Secondary Information:**	
**Order of Authors:**	Lukas F. K. Kuderna
	Chad Tomlinson
	LaDeana W. Hillier
	Annabel Tran
	Ian Fiddes
	Joel Armstrong
	Hafid Laayouni
	David Gordon
	John Huddleston
	Raquel Garcia Perez
	Inna Povolotskaya
	Aitor Serres Armero
	Jèssica Gómez Garrido
	Daniel Ho
	Paolo Ribeca
	Tyler Alioto
	Richard E. Green
	Benedict Paten
	Arcadi Navarro
	Jaume Betranpetit
	Javier Herrero
	Evan E. Eichler
	Andrew J. Sharp
	Lars Feuk
	Wesley C. Warren
	Tomas Marques-Bonet
**Order of Authors Secondary Information:**	
**Opposed Reviewers:**	
**Additional Information:**	
**Question**	**Response**
Are you submitting this manuscript to a	No

Powered by Editorial Manager® and ProduXion Manager® from Aries Systems Corporation

special series or article collection?	
**Experimental design and statistics**<br><br>Full details of the experimental design and statistical methods used should be given in the Methods section, as detailed in our Minimum Standards Reporting Checklist. Information essential to interpreting the data presented should be made available in the figure legends.<br><br>Have you included all the information requested in your manuscript?	Yes
**Resources**<br><br>A description of all resources used, including antibodies, cell lines, animals and software tools, with enough information to allow them to be uniquely identified, should be included in the Methods section. Authors are strongly encouraged to cite Research Resource Identifiers (RRIDs) for antibodies, model organisms and tools, where possible.<br><br>Have you included the information requested as detailed in our Minimum Standards Reporting Checklist?	Yes
**Availability of data and materials**<br><br>All datasets and code on which the conclusions of the paper rely must be either included in your submission or deposited in publicly available repositories (where available and ethically appropriate), referencing such data using a unique identifier in the references and in the "Availability of Data and Materials" section of your manuscript.<br><br>Have you have met the above requirement as detailed in our Minimum Standards Reporting Checklist?	Yes

Manuscript
Click here to download Manuscript Kuderna_et_al.MAIN.docx

A 3-way hybrid approach to generate a new high quality chimpanzee reference genome (Pan_tro_3.0)

Lukas F.K. Kuderna[1], Chad Tomlinson[2], LaDeana W. Hillier[2], Annabel Tran[3], Ian Fiddes[4], Joel Armstrong[4], Hafid Laayouni[1], David Gordon[5], John Huddleston[5], Raquel Garcia Perez[1], Inna Povolotskaya[1], Aitor Serres Armero[1], Jèssica Gómez Garrido[6], Daniel Ho[7], Paolo Ribeca[8], Tyler Alioto[6], Richard E. Green[9,12], Benedict Paten[4], Arcadi Navarro[1,6,10], Jaume Betranpetit[1], Javier Herrero[3], Evan E. Eichler[5], Andrew J. Sharp[7], Lars Feuk[11,*], Wesley C. Warren[2,*], Tomas Marques-Bonet[1,6,10*]

(1) Institut de Biologia Evolutiva, (CSIC-Universitat Pompeu Fabra), PRBB, Doctor Aiguader 88, Barcelona, Catalonia 08003, Spain.

(2) McDonnell Genome Institute, Department of Medicine, Department of Genetics, Washington University School of Medicine, St. Louis, MO 63108, USA.

(3) Bill Lyons Informatics Centre, UCL Cancer Institute, University College London, London, UK.

(4) Genomics Institute, University of California Santa Cruz and Howard Hughes Medical Institute, Santa Cruz, CA 95064, USA.

(5) Department of Genome Sciences, University of Washington School of Medicine, Seattle, WA 98195, USA.

(6) CNAG-CRG, Centre for Genomic Regulation (CRG), Baldiri i Reixac 4, 08028, Barcelona, Spain.

(7) Department of Genetics and Genomic Sciences, Icahn School of Medicine at Mount Sinai, New York, NY 10029, USA

(8) The Pirbright Institute, Ash Road, Pirbright, Woking, GU24 0NF, United Kingdom

(9) Department of Biomolecular Engineering, University of California Santa Cruz, 1156 High Street, Santa Cruz, CA 95060, USA.

(10) Institucio Catalana de Recerca i Estudis Avançats (ICREA), Barcelona, Catalonia 08010, Spain

(11) Department of Immunology, Genetics and Pathology, Science for Life Laboratory, Uppsala University, Uppsala, Sweden

(12) Dovetail Genomics, Santa Cruz, CA 95060, USA

Abstract

Background

The chimpanzee is arguably the most important species for the study of human origins. A key resource for these studies is a high quality reference genome assembly, however, as most mammalian genomes, the current iteration of the chimpanzee reference genome assembly it is highly fragmented. In the current iteration of the chimpanzees reference genome assembly (Pan_tro_2.1.4), the sequence is scattered across more then 183,000 contigs and incorporating over 159,000 gaps, with a genome wide contig N50 of 51 Kbp.

Findings

In this work we produce an extensive and diverse array of sequencing datasets to rapidly assemble a new chimpanzee reference that surpasses previous iterations in bases represented and organized in large scaffolds. To this end, we show substantial improvements over the current release of the chimpanzee genome (Pan_tro_2.1.4) by several metrics, such as: increased contiguity by >750% and 300% on contigs and scaffolds, respectively; closure of 77% of gaps in the Pan_tro_2.1.4 assembly gaps spanning >850 Kbp of novel coding sequence based on RNASeq data. We furthermore report over 2,700 genes that had putatively erroneous frame-shift predictions to human in Pan_tro_2.1.4 and show a substantial increase in the annotation of repetitive elements.

Conclusions

We apply a simple 3-way hybrid approach to considerably improve the reference genome assembly for the chimpanzee, providing a valuable resource to study human origins. We furthermore produced extensive sequencing datasets that are all derived from the same cell line, generating a broad non-human benchmark dataset.

Keywords

Chimpanzee reference genome, Assembly, Genomics

Data description

Creating a non-human sequencing benchmark dataset

To test the potentially combinatorial power of varied sequencing and mapping strategies, we created several different datasets on different platforms, all derived from a single male western chimpanzee (‘Clint’, Coriell identifier S006007), the same individual used to generate the current Chimpanzee genome assembly. We produced ~120-fold sequence coverage of overlapping 250 bps reads (~400 bps fragment) on the Illumina HiSeq 2500 platform; ~9-fold sequence coverage from 43 Pacific Biosciences SMRT-Cells with P5-C3 chemistry on the RSII instrument; Illumina TruSeq Synthetic long reads at around 2-fold coverage; 1 lane of *in vitro* proximity ligation read pairs (prepared as a Chicago library by Dovetail Genomics) sequenced on the Illumina HiSeq 2000 platform.

These diverse datasets complement the resources that were already available for the same cell line, namely 6-fold coverage of ABI Sanger capillary reads used for the initial chimpanzee genome assembly, a 100 bps paired Illumina HiSeq data, a fosmid library at 6-fold physical coverage with available end sequences, a BAC library at 3-

fold physical coverage with available end sequences and around 700 finished BACs [1]. Altogether, these data constitute an extensive and, to our knowledge, unprecedented non-human, non-model organism benchmarking dataset for different sequencing strategies.

Assembly generation

We generated a complete *de novo* assembly for the chimpanzee with a combination of the datasets. At each step of our assembly we measured increase in contiguity by means of the N50 statistic, which is defined as the length of a contig or scaffold such that 50% of the assembly bases are contained in contigs or scaffolds of at least that length. The starting point of our assembly scaffolding efforts are contigs generated with DISCOVAR *de novo* [2] from 250 bps paired end reads. These reads are derived from a 400 bps library, resulting in pairs that overlap over a ~50 bps region, a feature that is exploited by the assembler. While based on Illumina sequencing, these libraries have recently been shown to produce assemblies superior in contiguity when compared to assemblies derived from conventional Illumina libraries [3]. Our base assembly had a contig N50 of 87 Kbp, and was then scaffolded using proximity ligation read-pairs generated by the Chicago method [4] and sequenced on the Illumina platform. These data increased the N50 to 26 Mbp. Notably, individual scaffolds exceed lengths of 75 Mbp and therefore already reach the order of magnitude of full chromosomal arms. Despite a substantial gain in scaffold continuity, remaining gap structure required us to attempt closure with long-read single molecule sequences by PacBio using PBJelly [5]. By this means, we filled over 38,000 gaps (or 55%) among all scaffolds and in so doing increased the contig N50 by over 320% to 283 Kbp when compared to the base assembly (see Table 1). While

we went on to further improve the assembly with additional data (see below), these statistics give an approximation of the contiguity that can be expected for *de novo* assemblies of previously unsequenced species using our three-way hybrid approach: contigs derived from overlapping 250 bps paired end reads to scaffold with in vitro HiC, and fill remaining gaps with PacBio data. When the contiguity metrics of this intermediate assembly are compared to other representative non-human primate genomes (as annotated by NCBI Refseq category, July 1, 2016; see supplementary material), we observed superior connectivity in contig structure within our assembly compared to all others. The only exception is the gorilla genome, recently assembled from deep (~75-fold) long-read sequences [6]. However, our stepwise method offers an approach that is considerably cheaper.

Assembly refinement and comparison to Pan_tro_2.1.4

For the final release of the chimpanzee assembly, we created a reference assembly that leveraged previous resources generated from the same individual [1]. First, we merged in regions from Pan_tro_2.1.4 that were derived from Clint and gapped in our assembly. It is known that Pan_tro_2.1.4 contains sequences from different chimpanzees. To do so, we extracted flanking sequence regions of gaps in our assembly and mapped all to Pan_tro_2.1.4, keeping only unique and concordant mappings that do not span any gaps within Pan_tro_2.1.4, and merged the spanned Pan_tro_2.1.4 sequence in.

To ensure accuracy was not sacrificed for continuity gains we utilized various methods to measure error. Given that our assembly likely contained some erroneous links between contigs or misassembled contigs as a result of *de novo* assembly,

conformational mapping or merging mistakes, we first used discordant mapping of fosmid end sequences (~40 Kbp insert size) to identify any large misassemblies. We identified 17 such scaffold errors and manually broke apart each. We also sought to correct any remaining single base substitutions or small indels (<6 bps) with a series of custom mapping and base integration programs (see supplementary material). With the same Illumina data used to generate the first draft assembly (DISCOVAR *de novo*) we corrected more than 500,000 single base or indel errors. As another measure of quality we produced whole genome alignments to Pan_tro_2.1.4 and find our assembly aligns with on average 99.9% identity, and the magnitude of remaining differences can thus reasonably explained by the allelic diversity of western chimpanzees [7].

Our final assembly, named Pan_tro_3.0, spans 2.95 Gbp in ordered and oriented chromosomal sequences. An additional 140 Mbp of sequence is assigned to chromosomes, but their order and orientation unknown, and 123 Mbp remain of unknown chromosomal origin. Pan_tro_3.0 has a genome-wide contig and scaffold N50 of 385 Kbp and 27 Mbp, respectively, constituting an improvement in contiguity over Pan_tro_2.1.4 of 760% and 300%, respectively (see Figure 1a and Table1). We observed this increase across all non-finished chromosomes, with the most pronounced effect on the X chromosome (see Figure 1b). This chromosome shows the highest degree of fragmentation in Pan_tro_2.1.4, likely due to the fact that the effective sequence coverage on the sex chromosomes is only half that of the autosomes, namely around 3-fold in the original assembly. We increased the contig N50 on the X chromosome by 3,250% from 13 Kbp to 422 Kbp, thus bringing its contiguity to the range observed on autosomes.

Overall, we decreased the number of contigs by more than 60% from 183,860 to 72,226 and the number of gaps by 83% from 156,857 to 26,715. As gap structures between the assemblies may not correspond, we identified filled gaps from Pan_tro_2.1.4 by extracting their flanking regions and mapping them onto Pan_tro_3.0. By keeping only unique and concordant mappings that do not span any gaps in Pan_tro_3.0, we estimate the sequences of 122,943 (77%) gaps to be filled, amounting for 60.3 Mbp of sequence. The majority of these fill sequences are comparably short (see Figure 1C) and significantly enriched in interspersed genomic repeats with 58% of them ($p<0.0001$, feature permutation test) into repeats. Of these, around 16 Mbp are fully embedded within fill sequences corresponding to, amongst others, over 29,650 novel short interspersed nuclear elements (SINE) and 20,888 novel long interspersed nuclear elements (LINE) annotations.

Table 1 - Assembly statistics comparing the previous chimpanzee assembly, our intermediary assembly based on the 3-way hybrid and the finished assembly Pan_tro_3.0. In this context, we defined gaps at stretches of at least 10 consecutive "N" in the assembly. Contigs are defined as contiguous stretches of sequence without gaps.

	**Pan_tro_2.1.4**	**3-way hybrid (intermediary)**	**Pan_tro_3.0**
**Scaffold N50 (bps)**	8,925,874	26,681,610	26,972,556
**Contig N50 (bps)**	50,665	282,774	384,816
**Contig N90 (bps)**	7,231	41,655	53,112
**Assembly length (bps)**	3,309,577,923	2,992,696,208	3,231,154,112
**Assembly length w\o N's (bps)**	2,902,338,968	2,990,712,612	3,132,603,062
**Scaffolds**	24,129	45,000	44,448

**Contigs**	183,827	76,674	72,226
**Gaps**	159,698	31,674	26,715

Repeat resolution

Large genomic repeats constitute a major confounding factor in genome assembly and are therefore one of the main reasons for their fragmentation and thus, the assembly repeat representation can be a proxy of its quality. To assess the repeat resolution of interspersed repeats, we masked Pan_tro_3.0 using RepeatMasker [8] selecting chimpanzee specific repeats, resulting in 1.64 Gbp (52.2%) being annotated as repeats. The proportion of repetitive elements is similar in Pan_tro_2.1.4 (50.9%), however, given the large amount of newly resolved sequences this translates into a substantial increase in annotated repeats. Specifically, we annotate 164 Mbp of novel repeats in Pan_tro_3.0, comprising around 10% of the whole repeat annotation. We observe this increase consistently across all families of interspersed repeats (see Figure 1D). The increases range as high as 300% for satellite sequences, corresponding to an additional 68.2 Mbp of newly resolved sequence in this category. We also increased the amount of annotated SINE by 27.9 Mbp, including 83,637 additional resolved copies of *Alu* elements. We find the increase in annotations to be negatively correlated with age for *Alu* elements, and thus find the highest increase (8.8%) for the youngest and least divergent subfamily (*AluY*), suggesting that common high identity repeats are now better resolved. We furthermore added 38.2 Mbp of LINE to the assembly, corresponding to over 44,791 additional copies of L1 elements. We also observed a noteworthy increase in annotated long terminal repeats (LTR), adding 15.9 Mbp to this repeat category, corresponding to 30,574 additional annotated copies of endogenous retroviruses (ERV) in the genome. When comparing

all types of interspersed repeats between Pan_tro_2.1.4 and Pan_tro_3.0, we find a median increase of 4.7% of sequence, highlighting that repeat resolution is much improved in Pan_tro_3.0 (see supplementray table S4).

Representation of segmental duplications

To analyze the representation of segmental duplications in Pan_tro_3.0, we applied two alternative approaches: First, we performed a whole genome assembly comparison (WGAC) to compare repeat-free sequences of the assembly to itself. This method identifies duplicated sequence in blocks of at least 1 Kbp with 90% identity or higher. Excluding unplaced contigs, we find 140 Mbp of non-redundant duplicated sequence in Pan_tro_3.0 chromosomes, or 4.46% of the non-gap bases in the assembly, results that are consistent with previous read-depth estimates for chimpanzee [9] and analyses of high quality, finished human genome assemblies (see supplementary material S3). Second, we identified duplications by whole-genome shotgun sequence detection (WSSD) that identifies duplications at least 10 Kbp long with over 94% identity by detecting regions of increased read depth compared to known unique regions. We used 31,366,275 Sanger capillary reads derived from Clint, and find 51 Mbp of duplicated sequence meeting these criteria on placed chromosomes, compared to 68 Mbp detected by WGAC.

Genome wide, we discovered 178,245 redundant pairwise alignments corresponding to 388 Mbp of non-redundant sequence above 1Kbp in length and 90% identity (12.39% of the genome sequence excluding gaps) by WGAC, and 63 Mbp of duplicated sequence by WSSD (compared to 284 Mbp WGAC ≥10 Kbp, >94% identity). Altogether, these results suggest that segmental duplications are well resolved in Pan_tro_3.0 on the chromosomal level, however, we are likely to be

overestimating the total amount of segmental duplications genome wide by including an elevated rate of false-positive paralogous regions localized on unplaced scaffolds.

Gene annotation

We produced a new gene annotation based on projections from all human transcripts in the GENCODE annotation V24 set combined with RNA-seq data derived from brain, heart, liver and testis from three different individuals [10]. To quantify the effect of the underlying sequence on the annotation, we annotated Pan_tro_2.1.4. with the same data. We observe improvements in gene annotation in Pan_tro_3.0 in all considered metrics: We increased the number of recovered consensus gene models for protein coding transcripts by 2.7%, and are now able to project and annotate 89.5% of the GENCODE human coding transcripts onto the new assembly. The average coverage of these transcripts within the genome is 98.9%, a gain of 2%. We observe an increase of 6.6% in transcripts with multiple mappings, suggesting that paralogous coding duplications are better represented in this assembly. We checked for newly resolved exonic sequences in filled gaps with respect to Pan_tro_2.1.4, and find 17,818 exons, amounting to 851 Kbp of non-overlapping sequence to be fully embedded within them. Altogether, we retrieved models for 77,858 coding transcripts corresponding to the isoforms of 20,373 coding genes.

Perhaps most strikingly, we found 5,039 human coding transcripts corresponding to 2,728 genes with predicted frameshift mutations in Pan_tro_2.1.4 to human, but not in Pan_tro_3.0. Given that both assemblies are mainly based on data from the same individual, the majority of these predictions constitute putative sequence errors in Pan_tro_2.1.4.

In summary, we describe a hybrid assembly approach to obtain a more complete de novo chimpanzee reference genome assembly, substantially increasing contiguity metrics within it.

Figure 1

A: Genome wide distribution of contig lengths between Pan_tro_2.1.4 and Pan_tro_3.0. The peak for Pan_tro_3.0 is shifted to higher values by an order of magnitude.

B: Increase in contig N50 for all chromosomes that were not finished with clones in Pan_tro_2.1.4 or Pan_tro_3.0.

C: Length distribution of filled gaps in Pan_tro_3. Negative values constitute wrongly separated overlapping contig ends in Pan_tro_2.1.4.

D: Increase in annotated interspersed repeats separated by repeat family.

Declarations

Abbreviations

bps: base pairs, Kbp: kilo base pairs, Mbp: mega base pairs, indel: insetion-deletion, SINE: short interspersed nuclear element, LINE: long interspersed nuclear element, LTR: long terminal repeat, ERV: endogenous retrovirus, WGAC: whole genome assembly comparison, WSSD: whole-genome shotgun sequence detection.

Competing interests

REG is co-founder of Dovetail Genomics

Funding

LFKK is supported by an FPI fellowship associated to BFU2014-55090-P (FEDER); LF is supported by the Swedish Foundation for Strategic Research F06-0045 and the Swedish Research Council; EEE is an investigator of the Howard Hughes Medical Institute. AJS is supported by NIH grants DA033660, HG006696, HD073731 and MH097018, and research grant 6-FY13-92 from the March of Dimes TMB is supported by MINECO BFU2014-55090-P (FEDER), BFU2015-7116-ERC and BFU2015-6215-ERC, Fundacio Zoo Barcelona and Secretaria d'Universitats i Recerca del Departament d'Economia i Coneixement de la Generalitat de Catalunya. This work was supported, in part, by grants from the U.S. National Institutes of Health (NIH grants HG002385 to E.E.E., HG007990 and HG007234 to B.P.).

Author's Contributions

TMB, WCW and LF conceived the study; LFKK, CT, LWH and REG produced and analyzed the assembly; IF, JA, JGG, TA, BP, AT, HL, JB, RGP, IP, ASA, JHe, PR, DH, AN, and AJS produced, analyzed and interpreted the assembly and annotations; DG, JHu and EEE analyzed segmental duplications; TMB, WCW and LFKK wrote the manuscript with input from all authors.

Acknowledgements

We would like to acknowledge Bojan Obradovic and James Richardson for sequence contribution. The authors acknowledge the use of the UCL Legion High Performance Computing Facility (Legion@UCL), and associated support services, in the completion of this work.

Availability of supporting data

Supporting data area available through the GigaDB database. This Whole Genome Shotgun project has been deposited at DDBJ/ENA/GenBank under the accession AACZ00000000. The version described in this paper is version AACZ04000000. The assembly is available at https://www.ncbi.nlm.nih.gov/assembly/GCF_000001515.7 and at the UCSC genome browser under the identifier panTro5.

References

1. Mikkelsen TS, , Evan E. Eichler MCZ, Jaffe, David B., Yang S-P, , Wolfgang Enard IH, Bork, Peer, Butler J, Fronick, et al. Initial sequence of the chimpanzee genome and comparison with the human genome. Nature. 2005;437:69–87.

2. Weisenfeld NI, Yin S, Sharpe T, Lau B, Hegarty R, Holmes L, et al. Comprehensive variation discovery in single human genomes. Nat. Genet.; 2014;46:1350–5.

3. Chaisson MJP, Wilson RK, Eichler EE. Genetic variation and the de novo assembly of human genomes. Nat. Rev. Genet.; 2015;16:627–40.

4. Putnam NH, Connell BO, Stites JC, Rice BJ, Hartley PD, Sugnet CW, et al. Chromosome-scale shotgun assembly using an in vitro method for long-range linkage. Genome Res. 2016;1–25.

5. English AC, Richards S, Han Y, Wang M, Vee V, Qu J, et al. Mind the gap: upgrading genomes with Pacific Biosciences RS long-read sequencing technology. Liu Z, editor. PLoS One; 2012

6. Gordon D, Huddleston J, Chaisson MJP, Hill CM, Kronenberg ZN, Munson KM, et al. Long-read sequence assembly of the gorilla genome. Science;2016;0344.

7. Prado-Martinez J, Sudmant PH, Kidd JM, Li H, Kelley JL, Lorente-Galdos B, et al. Great ape genetic diversity and population history. Nature. 2013;499:471–5.

8. Smit A, Hubley R, Green P. RepeatMasker Open-3.0. RepeatMasker. 1996. p. www.repeatmasker.org.

9. Cheng Z, Ventura M, She X, Khaitovich P, Graves T, Osoegawa K, et al. A genome-wide comparison of recent chimpanzee and human segmental duplications. Nature. 2005;437:88–93.

10. Ruiz-Orera J, Hernandez-Rodriguez J, Chiva C, Sabidó E, Kondova I, Bontrop R, et al. Origins of De Novo Genes in Human and Chimpanzee. Noonan J, editor. PLOS Genet.;2015;11:e1005721.

Figure

Click here to download Figure Kuderna_et_al_Figure_1.pdf

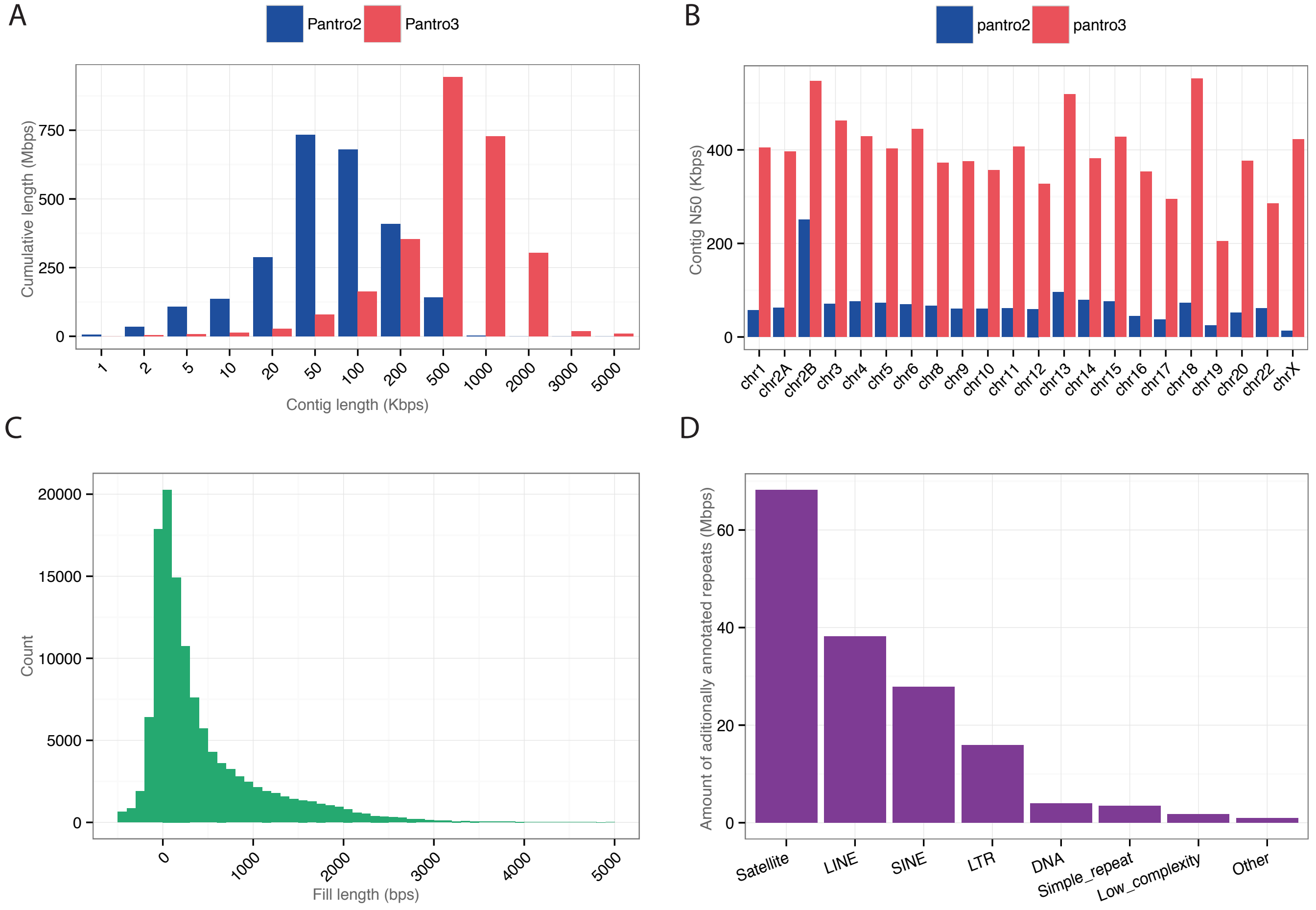

Click here to access/download
Supplementary Material
Kuderna_et_al.SUPPLEMENTARY.docx

description of data to submit

Click here to access/download
Supplementary Material
DATA_SUBMISSION_FILE
