# Supplementary material for: A 3-way hybrid approach to generate a new high-quality chimpanzee reference genome (Pan_tro_3.0)
Source: Gigascience. 2017 Oct 30;6(11):1–6. doi: 10.1093/gigascience/gix098 (PMC5714192; doi:10.1093/gigascience/gix098)
Supplement: GIGA-D-16-00140_Revision-1.pdf [file gix098_giga-d-16-00140_revision-1.pdf]

GigaScience

A 3-way hybrid approach to generate a new high quality chimpanzee reference genome (Pan_tro_3.0)

--Manuscript Draft--

**Manuscript Number:**	GIGA-D-16-00140R1
**Full Title:**	A 3-way hybrid approach to generate a new high quality chimpanzee reference genome (Pan_tro_3.0)
**Article Type:**	Data Note

Funding Information:

Ministerio de Economía y Competitividad (BFU2014-55090-P)	Mr Tomas Marques-Bonet
Stiftelsen för Strategisk Forskning (F06-0045)	Mr Lars Feuk
National Institutes of Health (DA033660)	Mr Andrew J. Sharp
National Institutes of Health (HG006696)	Mr Andrew J. Sharp
National Institutes of Health (HD073731)	Mr Andrew J. Sharp
National Institutes of Health (MH097018)	Mr Andrew J. Sharp
March of Dimes Foundation (6-FY13-92)	Mr Andrew J. Sharp
Ministerio de Economía y Competitividad (BFU2015-7116-ERC)	Mr Tomas Marques-Bonet
Ministerio de Economía y Competitividad (BFU2015-6215-ERC)	Mr Tomas Marques-Bonet
National Institutes of Health (HG002385)	Mr Evan E. Eichler
National Institutes of Health (HG007990)	Mr Benedict Paten
National Institutes of Health (HG007234)	Mr Benedict Paten

Abstract:

Background:
The chimpanzee is arguably the most important species for the study of human origins. A key resource for these studies is a high quality reference genome assembly, however, as most mammalian genomes, the current iteration of the chimpanzee reference genome assembly it is highly fragmented. In the current iteration of the chimpanzees reference genome assembly (Pan_tro_2.1.4), the sequence is scattered across more then 183,000 contigs and incorporating over 159,000 gaps, with a genome wide contig N50 of 51 Kbp.

Findings:
In this work we produce an extensive and diverse array of sequencing datasets to rapidly assemble a new chimpanzee reference that surpasses previous iterations in bases represented and organized in large scaffolds. To this end, we show substantial improvements over the current release of the chimpanzee genome (Pan_tro_2.1.4) by several metrics, such as: increased contiguity by >750% and 300% on contigs and scaffolds, respectively; closure of 77% of gaps in the Pan_tro_2.1.4 assembly gaps spanning >850 Kbp of novel coding sequence based on RNASeq data. We furthermore report over 2,700 genes that had putatively erroneous frame-shift predictions to human in Pan_tro_2.1.4 and show a substantial increase in the annotation of repetitive elements.

Conclusions:
We apply a simple 3-way hybrid approach to considerably improve the reference genome assembly for the chimpanzee, providing a valuable resource to study human origins. We furthermore produced extensive sequencing datasets that are all derived from the same cell line, generating a broad non-human benchmark dataset.

Powered by Editorial Manager® and ProduXion Manager® from Aries Systems Corporation

**Corresponding Author:**	Lukas F. K. Kuderna<br><br>SPAIN
**Corresponding Author Secondary Information:**	
**Corresponding Author's Institution:**	
**Corresponding Author's Secondary Institution:**	
**First Author:**	Lukas F. K. Kuderna
**First Author Secondary Information:**	
**Order of Authors:**	Lukas F. K. Kuderna
	Chad Tomlinson
	LaDeana W. Hillier
	Annabel Tran
	Ian Fiddes
	Joel Armstrong
	Hafid Laayouni
	David Gordon
	John Huddleston
	Raquel Garcia Perez
	Inna Povolotskaya
	Aitor Serres Armero
	Jèssica Gómez Garrido
	Daniel Ho
	Paolo Ribeca
	Tyler Alioto
	Richard E. Green
	Benedict Paten
	Arcadi Navarro
	Jaume Betranpetit
	Javier Herrero
	Evan E. Eichler
	Andrew J. Sharp
	Lars Feuk
	Wesley C. Warren
	Tomas Marques-Bonet
**Order of Authors Secondary Information:**	
**Response to Reviewers:**	Dear Dr. Hans Zauner,<br><br>Please find attached the resubmission of our revised manuscript GIGA-D-16-00140; A 3-way hybrid approach to generate a new high quality chimpanzee reference genome (Pan_tro_3.0), together with a point by point response of the comments of all

reviewers. We believe that we satisfactorily address all the comments in there.

We also wanted to report an issue that has been brought to us after a revision on the assembly. We have detected several small inversions affecting either full contigs within a scaffold, or the end of a contig. Because these events are predominantly rather small, they have previously escaped our notice when assessing (large scale) structural errors using clones, as they are only detectable by fine scale comparisons to the previous chimpanzee assembly, as well as the human genome assembly. Nevertheless, in the spirit of full disclosure, we did not want to resubmit our revised manuscript without addressing the issue first, now included in the final version of the assembly. Altogether, this affects 2,990 fully inverted contigs, amounting for 20.5 Mb of sequence. These were flipped and left in place in a new version of the assembly. Furthermore, there are 1,505 pieces of contigs, where the breakpoint of the inversion lays within the contigs, amounting for 11.1 Mb of sequence. In these cases, we manually went through the flips and decided to move some off to the unplaced portion of the assembly, and inverted some in place, depending on how clearly we could assign them to belonging to the chromosome in question. Cumulatively, there are now 31.6Mb of sequence in the assembly were we have changed the orientation. This affects 1,938 of coding exons amounting for 276,641 bp of coding sequence. Please note that the overall sequence content of the assembly has remained the same.

We are currently in the process of resubmitting our revised assembly to NCBI. For the paper, we have chosen to explicitly state which version of the NCBIs accessions system is being used.

We are looking forward to your reply.

Kind Regards,

Lukas Kuderna & Tomas Marques-Bonet

########

#Please note we are attaching a formatted document for the reviews with all graphics in place. Below you will find the copied text only from that document.

Point by point rebuttal:

Reviewer 1:
Reviewer #1: The authors present several large new datasets of chimpanzee genome sequencing data, and they combine these datasets into a novel, high-quality genome assembly of Pan troglodytes. As the authors state, this is a valuable addition to the set of available genome sequence resources and a vast improvement in genome quality over the existing Pan troglodytes assembly. The manuscript needs some editing and cleaning up, but overall I believe it represents a significant contribution to the field and should eventually be published.

In the "Data description" section, the paper gives an overview of the datasets the authors used. This section would benefit from a clear introduction and description of the sequencing strategies they employed to process these datasets. I suggest that a new figure, in the form of a simple flowchart, could be a helpful visual aid: it would describe the assembly methods that were used to combine the various types of sequencing libraries, and it would illustrate the process of creating the 3-way hybrid intermediary assembly as well as the final (3.0) assembly. Additionally, the "Data description" section is mostly devoid of citations. More citations should be added in order to give proper attribution to the developers of the assembly methods, and to enable the reader to seek more information.

We have sought to clarify on the different sequencing strategies, and have added references where adequate in this section (Goodwin S et al. ,2016; Kuleshov et al, 2014; Putnam et al, 2016). We have also added the flowchart of the assembly process in supplementary figure 1 (see below). Nevertheless, we have only slightly modified

the introductory section, as it is our understanding that this is adequate for the data note format.

The authors discuss the sequence content they have added to the chimpanzee genome. It's interesting to see the length distribution of the gaps they have filled (Figure 1C), and I would be curious to see comparative length distributions for gaps they failed to fill, or for gaps they added.

We have added plots of length distributions for both of these cases. Supplementary figure S19 (first figure below) shows the length distribution of gaps we can identify as corresponding between Pantro_2.1.4 and Pan_tro_3, but fail to fill. Supplementary Figure S20 (second figure below) shows the length distribution of gaps present within Pan_tro_3.0. We note, that the overall shape of the distribution is similar, with peaks at small gap sizes.

The detail on the repeat resolution is also fascinating. I think the authors sell themselves short by noting that the repeat fraction of the assembly increases from 50.9% to 52.2%: given that they only increase the assembly sequence length by ~8%, this actually shows that most of the sequence they've added is repeat sequence, which is a useful indicator of the new assembly's added value. Similarly, Figure 1D, which shows the quantities of added repeat sequence for various repeat types, would be stronger if it also showed the quantities of already-existing repeat sequence for each type.

We have added a plot showing the full comparative repeat content of both Pan_tro2.1.4 and Pan_tro3 at supplementary Figure S21 (first figure below) , as well as a scaled version for repeat families with fewer annotations at Figure S22 (second figure below).

The authors compare the new (3.0) genome assembly to the existing (2.1.4) assembly. They observe a 99.9% overall sequence similarity and note that the 0.1% differences could be explained by SNPs; it would be interesting to see a deeper analysis of these SNPs, although this may be outside the scope of the manuscript.

While we agree that this would be an interesting exploration, we believe, in accordance with the reviewer, that an analysis of these SNPs is out of the scope of this manuscripts' format, especially considering its submission in the form of a GigaScience Data Note.

Also, in the section "Gene annotation", they note a large number of genes with frameshift mutations between the 2.1.4 assembly and the human genome assembly. This is striking, but a fully fair comparison would also mention the number of genes that also contain frameshift mutations (perhaps newly added frameshift mutations) in the 3.0 assembly.

We have now also included the count of frame-shift mutations specific for Pantro_3 for a fully fair comparison in this section (674). We have furthermore clarified, that these frame shifts are not necessarily due to sequence errors in Pan_tro_2.1.4, but might

Powered by Editorial Manager® and ProduXion Manager® from Aries Systems Corporation

also constitute allelic variation, as the assembly only randomly captures one of two alleles at a given locus.

The conclusion is strong, but it would be stronger with some additional context that describes the achievement in this manuscript. The genome assembly is higher quality. But is it also more efficient, or more economic? Have the authors innovated any new genome assembly methods? Have they demonstrated a technique that could be easily applied to other genome assemblies?

We clarify that our approach should easily be applicable to genomes of similar complexity. Nevertheless, we would like to refrain to comment on efficiency or economical value of the assembly for two reasons: First, the price for sequencing on several platforms has been shown to be extremely dependent on the time of sequence production, and has even dramatically decreased within the timeframe of this manuscript. Second, efficiency in the context of genome assembly is a rather subjective issue, as it not straightforward to decide what to measure efficiency against.

Minor errors:

Section "Assembly generation": "These reads are derived from a 400 bps library, resulting in pairs that overlap over a ~50 bps region". If a 400-bp fragment is sequenced to 250 bp from both ends, wouldn't that result in an overlap of ~100 bp rather than ~50 bp?

We thank the reviewer for catching this error, it is now corrected. The library was size selected to around 450bp, resulting in an overlap of around 50 bp when sequencing 250bp on each side.

Section "Assembly generation": "we observed superior connectivity". The word "connectivity" is unclear in this context; it might be better to simply repeat "contiguity".

We have clarified this sentence by rephrasing it.

Section "Repeat resolution": "We furthermore added 38.2 Mbp of LINE to the assembly, corresponding to over 44,791 additional copies of L1 elements." First of all, this should say "LINEs" rather than "LINE". Secondly, these numbers do not add up. A typical L1 element is 6 Kbp in length; thus, 44,791 copies of L1 elements should necessarily occupy over 260 Mbp of sequence.

We have corrected this flaw by leaving out that number. We erroneously counting L1 annotations, that do not necessarily correspond to fully resolved copies of L1 elements, as repeatmasker annotates partial matches of repetitive elements.

Section "Resolution of segmental duplications": This section should contain more citations, especially for the WGAC and WSSD methods, which are named but not described at all.

We have added references to both these methods in the corresponding section (Bailey et al. 2001; Bailey et al 2002).

Reviewer #2:

The genome of a chimpanzee is an important asset for the study of human evolution, and an high quality reference assembly is long overdue. The authors were able to significantly improve on the previous assemblies. I have no problem with the 3-way hybrid approach they have taken. The paper is written very clearly, and is fully appropriate for the data note format. My recommendation is that this manuscript is accepted without reservation.

Powered by Editorial Manager® and ProduXion Manager® from Aries Systems Corporation

We would like to thank the reviewer for the positive feedback.

Reviewer 3:

1. Lines 191 to 212. Is this an exploration of segmental duplications in the genome or over-representation and under-representation in the assembly? What is the support for the conclusion that “segmental duplications are well resolved.” The statement of the conclusion is very confusing: “we are likely to be overestimating ... by including an elevated rate of false positive paralogous regions…”

In this section, we explore the representation of segmental duplications within our assembly. We sought to clarify the confusion by rephrasing the concluding sentences of the paragraph to the following:

We then compared Pant_tro_3.0 to the human reference genome assembly GRCh38, an assembly that is based on a BAC hierarchical shotgun assembly strategy and may therefore be considered of gold standard with respect to representation of segmental duplications. We note similar proportions of bases in segmental duplications on chromosomal scaffolds (4,46% in Pan_tro_3.0 vs. 5,56% in GRCh38), however, we note an elevated genome wide rate of bases in duplications when including unplaced and unlocalized scaffolds, suggesting that our assembly includes false-positive paralogous regions within them (see supplementary Table 1).

By this means, we hope to clarify the questions of the reviewer: Our previous statements about segmental duplications being ‘well resolved’ referred to the comparable number of bases in segmental duplications between the Chimp and the Human assembly.

2. Lines 229 to 233. The finding is called “most striking” but it is accompanied by weak interpretation (“majority of … putative”). A little more investigation would probably support a strong claim of improvement. To estimate the veracity of the old frameshifts, clarify in what sense both assemblies are “mainly” based on data from the same individual, and measure if any frameshifted genes relied on reads from another individual. To estimate veracity of the new assemblies of these genes, rule out allelism in spanning reads, count framehshifts relative to human present in both assemblies, and count frameshifts exclusively in the new assembly. Presumably these counts are low.

We have now toned down this claim. We clarify to what extend the assemblies are based on the genomes of the same individual, and what proportions are derived from a different in Pan_tro_2.1.4 . We furthermore clarify, that these frameshifts don’t necessarily constitute fixed changes, but might also be due to allelic variation. Furthermore, we have included the number of genes with predicted frameshifts only in Pan_tro_3, but not in Pan_tro_2.1.4

3. Line 125 “17 scaffold errors”. Extrapolate the overall structural error rate using the number of bases spanned by fosmids. Extrapolate the likely number of remaining structural errors. Of errors fixed, where they attributable to the contig or the scaffold process specifically? Were the errors near particularly repetitive sequence?

Out of 671,716 fosmids with available end sequences for both ends from the CHORI-1251 library, 545,788 (81%) mapped with both ends and high quality (mismapping rate < 0.00001, MQ<=60) onto the same scaffold. Out of these, we find 539,315 (99%) to map with both ends in concordant orientation, and 6,473 (1%) with both ends in the discordant orientation. Cumulatively, these concordant mappings cover 2.7 Gbps (85%) of the whole assembly length. As PBJelly changes the naming scheme of the sequences after filling gaps, we could not deduce at which stage the structural errors we fixed where introduced.

4. Line 129 and 232 “500,000 SNPs … frameshift”. Explanation, investigation, or speculation would help. What caused SNPs in contigs relative to the Illumina reads used to create the base assembly? What caused frameshifts in the prior assembly?

Powered by Editorial Manager® and ProduXion Manager® from Aries Systems Corporation

We now try to clarify by speculating that most of these corrected errors are due to regions where PacBio data was incorporated into the assembly (line 129). Given the relatively low coverage of PacBio data, we did not apply self correction on the PacBio reads, but rather corrected the assembly after filling in gaps. This leads to an elevated rate of residual errors in regions derived from PacBio data. We hypothesize that comparatively few of these corrected errors lay within regions derived from Illumina data. Given the constant change of coordinate system between the assemblies (with each incorporated platform) it is not straightforward to know which region in the assembly is finally derived from what platform.
We clarify that frameshifts with respect to Pantro2.1.4 are either because of allelic variation or sequencing errors in the Sanger data used to assemble Pantro2.1.4.

5. Line 65 “SMRTcells … synthetic long reads”. The manuscript does not address issues of integrating these technologies. Did either require error correction? Was there ever any overlap or disagreement between these two types of long reads? Was either more helpful than the other?

We had included an analysis of gap-filling performance and repeat resolution for PacBio and TruSeq SLR in the supplementary section S2, where we compare how well gaps in Pantro-2.1.4 are resolved using only either technology, as well as a combination of both. We did not incorporate the Truseq SLR data into the assembly based on the observed high rate of repeat collapse (see supplementary sections S2, supplementary Figures S2 and S3). Indeed, we see that many common high identity repeats are under-represented in sequencing data derived from this platform. We did not pre-correct the PacBio data, but rather run a post-assembly error correction, as described in the manuscript.

6. Line 223 “paralogous coding duplications are better represented”. What was the read coverage of these regions? Are these duplications specific to the chimp lineage or ancestral to primates? How is a paralogous coding duplication different from other kinds?

We now refrain to claim that, paralogous coding duplications are better represented’ as although we observe a shift to higher read depths in these regions, some of the newly added paralogs do no validate by excessive read depth.
7. Line 145 “bringing its continuity to the range”. The X chromosome N50 (422K) is actually larger than the average (385K). If the old assembly had smaller contigs at X due to half coverage of the X chromosome, then why isn’t that factor at play in the new assembly?

We believe this to be the case mainly due to two reasons: First, following a back of the envelope calculation with the Lander-Waterman equation, there are about 5% of bases without a single read when sampling them at a 3X coverage. This relationship is non-linear with respect to coverage, and the number of unsampled bases drops to essentially 0 at a 30X coverage. Second, because of the initially poor assembly quality on the X, many BACs have been finished and integrated into the assembly. These BACs were also used for the final AGP creation of our assembly, boosting contiguity on this chromosome.

8. Line 126. Were SNPs concentrated in the gap fill regions? In the gaps filled with low-coverage long reads?

We speculate that this is most likely the case. However, given the constant change in coordinate system during the assembly, and also during the correction process itself, it is not straigthforward for us to keep track of the origin of each genomic region within the assembly.

9. Line 74 “finished BAC”. The BACs never get mentioned again. Do the old and new assemblies agree with the BACs?

We now clarify that these BACs were integrated into Pan_tro_2.1.4 as well as the finished version of Pan_tro_3. Thus, by definition, the final assemblies agree with the BACs

Powered by Editorial Manager® and ProduXion Manager® from Aries Systems Corporation

	10. Line 76 “unprecedented”. Is there any need to make this controversial claim?<br><br>We have rephrased the sentence to tone down the claim.<br><br>11. Lines 89, 98, 128 “base assembly”. The DISCOVAR assembly is referenced by several names, some of which I confused with 2.1.4. Assign it a name or number?<br><br>The DISCOVAR base assembly is now consistently refered to as ‚DISCOVAR base assembly’<br><br>12. Line 92 should clarify this is a scaffold N50.<br><br>We clarified this regards the scaffold N50.<br><br>13. Line 95 “remaining gap structure required us to”. What is a gap structure? In what way was a response required?<br><br>We have rephrased this sentence to make it clearer.
**Additional Information:**	
**Question**	**Response**
Are you submitting this manuscript to a special series or article collection?	No
**Experimental design and statistics**<br><br>Full details of the experimental design and statistical methods used should be given in the Methods section, as detailed in our Minimum Standards Reporting Checklist. Information essential to interpreting the data presented should be made available in the figure legends.<br><br>Have you included all the information requested in your manuscript?	Yes
**Resources**<br><br>A description of all resources used, including antibodies, cell lines, animals and software tools, with enough information to allow them to be uniquely identified, should be included in the Methods section. Authors are strongly encouraged to cite Research Resource Identifiers (RRIDs) for antibodies, model organisms and tools, where possible.<br><br>Have you included the information requested as detailed in our Minimum Standards Reporting Checklist?	Yes
**Availability of data and materials**	Yes

Powered by Editorial Manager® and ProduXion Manager® from Aries Systems Corporation

All datasets and code on which the conclusions of the paper rely must be either included in your submission or deposited in publicly available repositories (where available and ethically appropriate), referencing such data using a unique identifier in the references and in the "Availability of Data and Materials" section of your manuscript.<br><br>Have you have met the above requirement as detailed in our Minimum Standards Reporting Checklist?	

Manuscript

Click here to download Manuscript Kuderna_et_al.MAIN_resubmission_ED.docx

A 3-way hybrid approach to generate a new high quality chimpanzee reference genome (Pan_tro_3.0)

Lukas F.K. Kuderna[1,2], Chad Tomlinson[3], LaDeana W. Hillier[3], Annabel Tran[4], Ian Fiddes[5], Joel Armstrong[5], Hafid Laayouni[1,6], David Gordon[7], John Huddleston[7], Raquel Garcia Perez[1], Inna Povolotskaya[1], Aitor Serres Armero[1], Jèssica Gómez Garrido[2], Daniel Ho[8], Paolo Ribeca[9], Tyler Alioto[2], Richard E. Green[10,11], Benedict Paten[5], Arcadi Navarro[1,2,12], Jaume Betranpetit[1], Javier Herrero[4], Evan E. Eichler[7], Andrew J. Sharp[8], Lars Feuk[13,*], Wesley C. Warren[3,*], Tomas Marques-Bonet[1,2,12*]

(1) Institut de Biologia Evolutiva, (CSIC-Universitat Pompeu Fabra), PRBB, Doctor Aiguader 88, Barcelona, Catalonia 08003, Spain.

(2) CNAG-CRG, Centre for Genomic Regulation (CRG), Baldiri i Reixac 4, 08028, Barcelona, Spain.

(3) McDonnell Genome Institute, Department of Medicine, Department of Genetics, Washington University School of Medicine, St. Louis, MO 63108, USA.

(4) Bill Lyons Informatics Centre, UCL Cancer Institute, University College London, London, UK.

(5) Genomics Institute, University of California Santa Cruz and Howard Hughes Medical Institute, Santa Cruz, CA 95064, USA.

(6) Bioinformatics Studies, ESCI-UPF, Pg. Pujades 1, 08003, Barcelona, Spain.

(7) Department of Genome Sciences, University of Washington School of Medicine, Seattle, WA 98195, USA.

(8) Department of Genetics and Genomic Sciences, Icahn School of Medicine at Mount Sinai, New York, NY 10029, USA

(9) The Pirbright Institute, Ash Road, Pirbright, Woking, GU24 0NF, United Kingdom

(10) Department of Biomolecular Engineering, University of California Santa Cruz, 1156 High Street, Santa Cruz, CA 95060, USA.

(11) Dovetail Genomics, Santa Cruz, CA 95060, USA

(12) Institucio Catalana de Recerca i Estudis Avançats (ICREA), Barcelona, Catalonia 08010, Spain

(13) Department of Immunology, Genetics and Pathology, Science for Life Laboratory, Uppsala University, Uppsala, Sweden

Abstract

Background

The chimpanzee is arguably the most important species for the study of human origins. A key resource for these studies is a high quality reference genome assembly, however, as most mammalian genomes, the current iteration of the chimpanzee reference genome assembly it is highly fragmented. In the current iteration of the chimpanzees reference genome assembly (Pan_tro_2.1.4), the sequence is scattered across more then 183,000 contigs and incorporating over 159,000 gaps, with a genome wide contig N50 of 51 Kbp.

Findings

In this work we produce an extensive and diverse array of sequencing datasets to rapidly assemble a new chimpanzee reference that surpasses previous iterations in bases represented and organized in large scaffolds. To this end, we show substantial improvements over the current release of the chimpanzee genome (Pan_tro_2.1.4) by several metrics, such as: increased contiguity by >750% and 300% on contigs and scaffolds, respectively; closure of 77% of gaps in the Pan_tro_2.1.4 assembly gaps spanning >850 Kbp of novel coding sequence based on RNASeq data. We furthermore report over 2,700 genes that had putatively erroneous frame-shift predictions to human in Pan_tro_2.1.4 and show a substantial increase in the annotation of repetitive elements.

Conclusions

We apply a simple 3-way hybrid approach to considerably improve the reference genome assembly for the chimpanzee, providing a valuable resource to study human origins. We furthermore produced extensive sequencing datasets that are all derived from the same cell line, generating a broad non-human benchmark dataset.

Keywords

Chimpanzee reference genome, Assembly, Genomics

Data description

Creating a non-human sequencing benchmark dataset

To test the potentially combinatorial power of varied sequencing and mapping strategies, we created several different datasets on different platforms, to try to leverage the advantages of each, as the shortcomings of one sequencing strategy might be compensated by another one [1]. All datasets are derived from a single male western chimpanzee ('Clint', Coriell identifier S006007), the same individual used to generate the current Chimpanzee genome assembly. We produced ~120-fold sequence coverage of overlapping 250 bps reads (~450 bps fragment) on the Illumina HiSeq 2500 platform, offering high accuracy and throughput, but comparatively short reads; ~9-fold sequence coverage from 43 Pacific Biosciences SMRT-Cells with P5-C3 chemistry on the RSII instrument, offering long reads at lower accuracy; Illumina TruSeq Synthetic long reads at around 2-fold coverage, offering long range information derived from local assemblies of ~10Kb fragments [2]; 1 lane of *in vitro* proximity ligation read pairs (prepared as a Chicago library by Dovetail Genomics)

[3] sequenced on the Illumina HiSeq 2000 platform, offering spatial contact information of the chromatin, that can be exploited for scaffolding.

These diverse datasets complement the resources that were already available for the same cell line, namely 6-fold coverage of ABI Sanger capillary reads used for the initial chimpanzee genome assembly, a 100 bps paired Illumina HiSeq data, a fosmid library at 6-fold physical coverage with available end sequences, a BAC library at 3-fold physical coverage with available end sequences and around 700 finished BACs [4]. Altogether, these data constitute an extensive non-human, and non-model organism benchmarking dataset for different sequencing strategies.

Assembly generation

We generated a complete *de novo* assembly for the chimpanzee with a combination of the datasets. At each step of our assembly we measured increase in contiguity by means of the N50 statistic, which is defined as the length of a contig or scaffold such that 50% of the assembly bases are contained in contigs or scaffolds of at least that length. The starting point of our assembly scaffolding efforts are contigs generated with DISCOVAR *de novo* [5] from 250 bps paired end reads. These reads are derived from a 450 bps library, resulting in pairs that overlap over a ~50 bps region, a feature that is exploited by the assembler. While based on Illumina sequencing, these libraries have recently been shown to produce assemblies superior in contiguity when compared to assemblies derived from conventional Illumina libraries [6]. The DISCOVAR base assembly had a contig N50 of 87 Kbp, and was then scaffolded using proximity ligation read-pairs generated by the Chicago method [3] and sequenced on the Illumina platform. These data increased the scaffold N50 to 26 Mbp. Notably, individual scaffolds exceed lengths of 75 Mbp and therefore already

reach the order of magnitude of full chromosomal arms. We sought to take advantage of this highly contiguous scaffolds and attempt closure of remaining gaps with long-read single molecule sequences by PacBio using PBJelly (PBJelly, RRID:SCR_012091) [7]. By this means, we filled over 38,000 gaps (or 55%) among all scaffolds and in so doing increased the contig N50 by over 320% to 283 Kbp when compared to the DISCOVAR base assembly (see Table 1). While we went on to further improve the assembly with additional data (see below), these statistics give an approximation of the contiguity that can be expected for *de novo* assemblies of previously unsequenced species using our three-way hybrid approach: contigs derived from overlapping 250 bps paired end reads to scaffold with in vitro HiC, and fill remaining gaps with PacBio data. When the contiguity metrics of this intermediate assembly are compared to other representative non-human primate genomes (as annotated by NCBI Refseq category, July 1, 2016; see supplementary material), we observed superior contiguity in contig structure within our assembly compared to all others. The only exception is the gorilla genome, recently assembled from deep (~75-fold) long-read sequences [8]. However, our stepwise method offers an approach that is considerably cheaper.

Assembly refinement and comparison to Pan_tro_2.1.4

For the final release of the chimpanzee assembly, we created a reference assembly that leveraged previous resources generated from the same individual [4]. First, we merged in regions from Pan_tro_2.1.4 that were derived from Clint and gapped in our assembly. It is known that Pan_tro_2.1.4 contains sequences from different chimpanzees. To do so, we extracted flanking sequence regions of gaps in our

assembly and mapped all to Pan_tro_2.1.4, keeping only unique and concordant mappings that do not span any gaps within Pan_tro_2.1.4, and merged the spanned Pan_tro_2.1.4 sequence in.

To ensure accuracy was not sacrificed for continuity gains we utilized various methods to measure error. Given that our assembly likely contained some erroneous links between contigs or misassembled contigs as a result of *de novo* assembly, conformational mapping or merging mistakes, we first used discordant mapping of fosmid end sequences (~40 Kbp insert size) to identify any large misassemblies. We identified 17 such scaffold errors and manually broke apart each. We also sought to correct any remaining single base substitutions or small indels (<6 bps) with a series of custom mapping and base integration programs (see supplementary material). With the same Illumina data used to generate the DISCOVAR base assembly, we corrected more than 500,000 single base or indel errors. Most of these residual errors are presumably derived from regions where PacBio data was incorporated into the assembly, as this platform is known to have an elevated error rate. As another measure of quality we produced whole genome alignments to Pan_tro_2.1.4 and find our assembly aligns with on average 99.9% identity, and the magnitude of remaining differences can thus reasonably explained by the allelic diversity of western chimpanzees [9].

For our final assembly, named Pan_tro_3.0, we integrated previously available finished clone sequences derived from Clint where possible. Pan_tro_3.0 spans 2.95 Gbp in ordered and oriented chromosomal sequences. An additional 140 Mbp of sequence is assigned to chromosomes, but their order and orientation unknown, and 123 Mbp remain of unknown chromosomal origin. Pan_tro_3.0 has a genome-wide contig and scaffold N50 of 385 Kbp and 27 Mbp, respectively, constituting an

improvement in contiguity over Pan_tro_2.1.4 of 760% and 300%, respectively (see Figure 1a and Table1). We observed this increase across all non-finished chromosomes, with the most pronounced effect on the X chromosome (see Figure 1b). This chromosome shows the highest degree of fragmentation in Pan_tro_2.1.4, likely due to the fact that the effective sequence coverage on the sex chromosomes is only half that of the autosomes, namely around 3-fold in the original assembly. We increased the contig N50 on the X chromosome by 3,250% from 13 Kbp to 422 Kbp, thus bringing its contiguity to the range observed on autosomes.

Overall, we decreased the number of contigs by more than 60% from 183,860 to 72,226 and the number of gaps by 83% from 156,857 to 26,715. As gap structures between the assemblies may not correspond, we identified filled gaps from Pan_tro_2.1.4 by extracting their flanking regions and mapping them onto Pan_tro_3.0. By keeping only unique and concordant mappings that do not span any gaps in Pan_tro_3.0, we estimate the sequences of 122,943 (77%) gaps to be filled, amounting for 60.3 Mbp of sequence. The majority of these fill sequences are comparably short (see Figure 1C) and significantly enriched in interspersed genomic repeats with 58% of them ($p<0.0001$, feature permutation test) into repeats. Of these, around 16 Mbp are fully embedded within fill sequences corresponding to, amongst others, over 29,650 novel short interspersed nuclear elements (SINE) annotations and 20,888 novel long interspersed nuclear elements (LINE) annotations.

Table 1 - Assembly statistics comparing the previous chimpanzee assembly, our intermediary assembly based on the 3-way hybrid and the finished assembly Pan_tro_3.0. In this context, we defined gaps at stretches of at least 10 consecutive "N" in the assembly. Contigs are defined as contiguous stretches of sequence without gaps.

	**Pan_tro_2.1.4**	**3-way hybrid (intermediary)**	**Pan_tro_3.0**
**Scaffold N50 (bps)**	8,925,874	26,681,610	26,972,556
**Contig N50 (bps)**	50,665	282,774	384,816
**Contig N90 (bps)**	7,231	41,655	53,112
**Assembly length (bps)**	3,309,577,923	2,992,696,208	3,231,154,112
**Assembly length w\o N's (bps)**	2,902,338,968	2,990,712,612	3,132,603,062
**Scaffolds**	24,129	45,000	44,448
**Contigs**	183,827	76,674	72,226
**Gaps**	159,698	31,674	26,715

Repeat resolution

Large genomic repeats constitute a major confounding factor in genome assembly and are therefore one of the main reasons for their fragmentation and thus, the assembly repeat representation can be a proxy of its quality. To assess the repeat resolution of interspersed repeats, we masked Pan_tro_3.0 using RepeatMasker (RepeatMasker, RRID:SCR_012954) [10] selecting chimpanzee specific repeats, resulting in 1.64 Gbp (52.2%) being annotated as repeats. The proportion of repetitive elements is similar in Pan_tro_2.1.4 (50.9%), however, given the large amount of newly resolved sequences this translates into a substantial increase in annotated repeats. Specifically, we annotate 164 Mbp of novel repeats in Pan_tro_3.0, comprising around 10% of the

whole repeat annotation. We observe this increase consistently across all families of interspersed repeats (see Figure 1D). The increases range as high as 300% for satellite sequences, corresponding to an additional 68.2 Mbp of newly resolved sequence in this category. We also increased the amount of annotated SINE by 27.9 Mbp, including 83,637 additional resolved copies of *Alu* elements. We find the increase in annotations to be negatively correlated with age for *Alu* elements, and thus find the highest increase (8.8%) for the youngest and least divergent subfamily (*AluY*), suggesting that common high identity repeats are now better resolved. We furthermore added 38.2 Mbp of sequence annotated as LINEs to the assembly. We also observed a noteworthy increase in annotated long terminal repeats (LTR), adding 15.9 Mbp to this repeat category, corresponding to 30,574 additional annotations of endogenous retroviruses (ERV) in the genome. When comparing all types of interspersed repeats between Pan_tro_2.1.4 and Pan_tro_3.0, we find a median increase of 4.7% of sequence, highlighting that repeat resolution is much improved in Pan_tro_3.0 (see supplementray table S4).

Representation of segmental duplications

To analyze the representation of segmental duplications in Pan_tro_3.0, we applied two alternative approaches: First, we performed a whole genome assembly comparison (WGAC) to compare repeat-free sequences of the assembly to itself [11]. This method identifies duplicated sequence in blocks of at least 1 Kbp with 90% identity or higher. Excluding unplaced contigs, we find 140 Mbp of non-redundant duplicated sequence in Pan_tro_3.0 chromosomes, or 4.46% of the non-gap bases in the assembly, results that are consistent with previous read-depth estimates for chimpanzee [12] and analyses of high quality, finished human genome assemblies (see supplementary material S3). Second, we identified duplications by whole-

genome shotgun sequence detection (WSSD) that identifies duplications at least 10 Kbp long with over 94% identity by detecting regions of increased read depth compared to known unique regions [13]. We used 31,366,275 Sanger capillary reads derived from Clint, and find 51 Mbp of duplicated sequence meeting these criteria on placed chromosomes, compared to 68 Mbp detected by WGAC.

Genome wide, we discovered 178,245 redundant pairwise alignments corresponding to 388 Mbp of non-redundant sequence above 1Kbp in length and 90% identity (12.39% of the genome sequence excluding gaps) by WGAC, and 63 Mbp of duplicated sequence by WSSD (compared to 284 Mbp WGAC ≥10 Kbp, >94% identity). We then compared Pant_tro_3.0 to the human reference genome assembly GRCh38, an assembly that is based on a BAC hierarchical shotgun assembly strategy and may therefore be considered of gold standard with respect to representation of segmental duplications. We note similar proportions of bases in segmental duplications on chromosomal scaffolds (4,46% in Pan_tro_3.0 vs. 5,56% in GRCh38), however, we note an elevated genome wide rate of bases in duplications when including unplaced and unlocalized scaffolds. This suggests that our assembly includes false-positive paralogous regions within them (see supplementary Table 1).

Gene annotation

We produced a new gene annotation based on projections from all human transcripts in the GENCODE annotation V24 set combined with RNA-seq data derived from brain, heart, liver and testis from three different individuals [14]. To quantify the effect of the underlying sequence on the annotation, we annotated Pan_tro_2.1.4. with the same data. We observe improvements in gene annotation in Pan_tro_3.0 in all considered metrics: We increased the number of recovered consensus gene models for protein coding transcripts by 2.7%, and are now able to project and annotate 89.5% of

the GENCODE human coding transcripts onto the new assembly. The average coverage of these transcripts within the genome is 98.9%, a gain of 2%. We also observe an increase of 6.6% in transcripts with multiple mappings. We checked for newly resolved exonic sequences in filled gaps with respect to Pan_tro_2.1.4, and find 17,818 exons, amounting to 851 Kbp of non-overlapping sequence to be fully embedded within them. Altogether, we retrieved models for 77,858 coding transcripts corresponding to the isoforms of 20,373 coding genes.

We find 5,039 human coding transcripts corresponding to 2,660 genes with predicted frameshift mutations in Pan_tro_2.1.4 to human, but not in Pan_tro_3.0. Conversely, we find 674 genes with predicted frameshift mutations to human that are present in Pan_tro_3, but not in Pan_tro_2.1.4. Given that both assemblies are mainly based on data from the same individual (with the exception of chromosome 21 and around 28% of chromosome 7 in Pan_tro_2.1.4, which where derived from a different individual), the majority of these predictions constitute either allelic variation or putative sequence errors in Pan_tro_2.1.4.

In summary, we describe a hybrid assembly approach to obtain a more complete de novo chimpanzee reference genome assembly, substantially increasing contiguity metrics within it. Our proposed assembly method should be easily applicable to different organisms of similar genomic architecture.

Figure 1

A: Genome wide distribution of contig lengths between Pan_tro_2.1.4 and Pan_tro_3.0. The peak for Pan_tro_3.0 is shifted to higher values by an order of

magnitude.

B: Increase in contig N50 for all chromosomes that were not finished with clones in Pan_tro_2.1.4 or Pan_tro_3.0.

C: Length distribution of filled gaps in Pan_tro_3. Negative values constitute wrongly separated overlapping contig ends in Pan_tro_2.1.4.

D: Increase in annotated interspersed repeats separated by repeat family.

Declarations

Abbreviations

bps: base pairs, Kbp: kilo base pairs, Mbp: mega base pairs, indel: insetion-deletion, SINE: short interspersed nuclear element, LINE: long interspersed nuclear element, LTR: long terminal repeat, ERV: endogenous retrovirus, WGAC: whole genome assembly comparison, WSSD: whole-genome shotgun sequence detection.

Competing interests

REG is co-founder of Dovetail Genomics

Funding

LFKK is supported by an FPI fellowship associated to BFU2014-55090-P (FEDER); LF is supported by the Swedish Foundation for Strategic Research F06-0045 and the Swedish Research Council; EEE is an investigator of the Howard Hughes Medical

Institute. AJS is supported by NIH grants DA033660, HG006696, HD073731 and MH097018, and research grant 6-FY13-92 from the March of Dimes TMB is supported by MINECO BFU2014-55090-P (FEDER), BFU2015-7116-ERC and BFU2015-6215-ERC, Fundacio Zoo Barcelona and Secretaria d'Universitats i Recerca del Departament d'Economia i Coneixement de la Generalitat de Catalunya. This work was supported, in part, by grants from the U.S. National Institutes of Health (NIH grants HG002385 to E.E.E., HG007990 and HG007234 to B.P.).

Author's Contributions

TMB, WCW and LF conceived the study; LFKK, CT, LWH and REG produced and analyzed the assembly; IF, JA, JGG, TA, BP, AT, HL, JB, RGP, IP, ASA, JHe, PR, DH, AN, and AJS produced, analyzed and interpreted the assembly and annotations; DG, JHu and EEE analyzed segmental duplications; TMB, WCW and LFKK wrote the manuscript with input from all authors.

Acknowledgements

We would like to acknowledge Bojan Obradovic and James Richardson for sequence contribution. The authors acknowledge the use of the UCL Legion High Performance Computing Facility (Legion@UCL), and associated support services, in the completion of this work.

Availability of supporting data

Supporting data are available through the GigaDB database (GigaDB, RRID:SCR_004002) [15]. This Whole Genome Shotgun project has been deposited at DDBJ/ENA/GenBank under the accession AACZ00000000. The version described in this paper is version AACZ04000000. The assembly is available at https://www.ncbi.nlm.nih.gov/assembly/GCF_000001515.7 and at the UCSC genome browser under the identifier panTro5. The assembly denominated Pan_tro_2.1.4 in the manuscript refers to Pan_troglodytes-2.1.4 with the RefSeq assembly accession GCF_000001515.6

References

1. Goodwin S, McPherson JD, McCombie WR. Coming of age: ten years of next-generation sequencing technologies. Nat Rev Genet [Internet]. Nature Publishing Group; 2016;17:333–51. Available from: https://www.ncbi.nlm.nih.gov/pubmed/27184599

2. Kuleshov V, Xie D, Chen R, Pushkarev D, Ma Z, Blauwkamp T, et al. Whole-genome haplotyping using long reads and statistical methods. Nat. Biotechnol. [Internet]. 2014;32:261–6. Available from: http://dx.doi.org/10.1038/nbt.2833

3. Putnam NH, Connell BO, Stites JC, Rice BJ, Hartley PD, Sugnet CW, et al. Chromosome-scale shotgun assembly using an in vitro method for long-range linkage. Genome Res. 2016;1–25.

4. Mikkelsen TS, , Evan E. Eichler MCZ, Jaffe, David B., Yang S-P, , Wolfgang Enard IH, Bork, Peer, Butler J, Fronick, et al. Initial sequence of the chimpanzee

genome and comparison with the human genome. Nature [Internet]. 2005 [cite;437:69–87. Available from: http://www.ncbi.nlm.nih.gov/pubmed/16136131

5. Weisenfeld NI, Yin S, Sharpe T, Lau B, Hegarty R, Holmes L, et al. Comprehensive variation discovery in single human genomes. Nat. Genet. [Internet]. Nature Publishing Group; 2014;46:1350–5. Available from: http://dx.doi.org/10.1038/ng.3121

6. Chaisson MJP, Wilson RK, Eichler EE. Genetic variation and the de novo assembly of human genomes. Nat. Rev. Genet. [Internet]. Nature Publishing Group; 2015;16:627–40. Available from: http://dx.doi.org/10.1038/nrg3933

7. English AC, Richards S, Han Y, Wang M, Vee V, Qu J, et al. Mind the gap: upgrading genomes with Pacific Biosciences RS long-read sequencing technology. Liu Z, editor. PLoS One [Internet]. Public Library of Science; 2012 ;7:e47768. Available from: http://dx.plos.org/10.1371/journal.pone.0047768

8. Gordon D, Huddleston J, Chaisson MJP, Hill CM, Kronenberg ZN, Munson KM, et al. Long-read sequence assembly of the gorilla genome. 2016;344.

9. Prado-Martinez J, Sudmant PH, Kidd JM, Li H, Kelley JL, Lorente-Galdos B, et al. Great ape genetic diversity and population history. Nature . 2013;499:471–5. Available from: http://www.nature.com/nature/journal/v499/n7459/full/nature12228.html#/accessions

10. Smit A, Hubley R, Green P. RepeatMasker Open-3.0. RepeatMasker. 1996. p. www.repeatmasker.org.

11. Bailey J a, Yavor AM, Massa HF, Trask BJ, Eichler EE. Segmental Duplications : Organization and Impact Within the Current Human Genome Project Assembly Segmental Duplications : Organization and Impact Within the Current Human Genome Project Assembly. Genome Res. 2001;11:1005–17.

12. Cheng Z, Ventura M, She X, Khaitovich P, Graves T, Osoegawa K, et al. A genome-wide comparison of recent chimpanzee and human segmental duplications. Nature. 2005;437:88–93.

13. Bailey J a, Gu Z, Clark R a, Reinert K, Samonte R V, Schwartz S, et al. Recent segmental duplications in the human genome. Science. 2002;297:1003–7.

14. Ruiz-Orera J, Hernandez-Rodriguez J, Chiva C, Sabidó E, Kondova I, Bontrop R, et al. Origins of De Novo Genes in Human and Chimpanzee. Noonan J, editor. PLOS Genet. [Internet]. Public Library of Science; 2015 ;11:e1005721. Available from: http://dx.plos.org/10.1371/journal.pgen.1005721

15. Kuderna LF, Tomlinson C, Hillier LW, Tran A, Fiddes I, Armstrong J et al. High quality chimpanzee reference genome (Pan_tro_3.0) from hybrid assembly approach. *GigaScience* Database. 2017**. http://dx.doi.org/10.5524/100327**

Figure 1

Click here to download Figure Kuderna_et_al_Figure_1.pdf

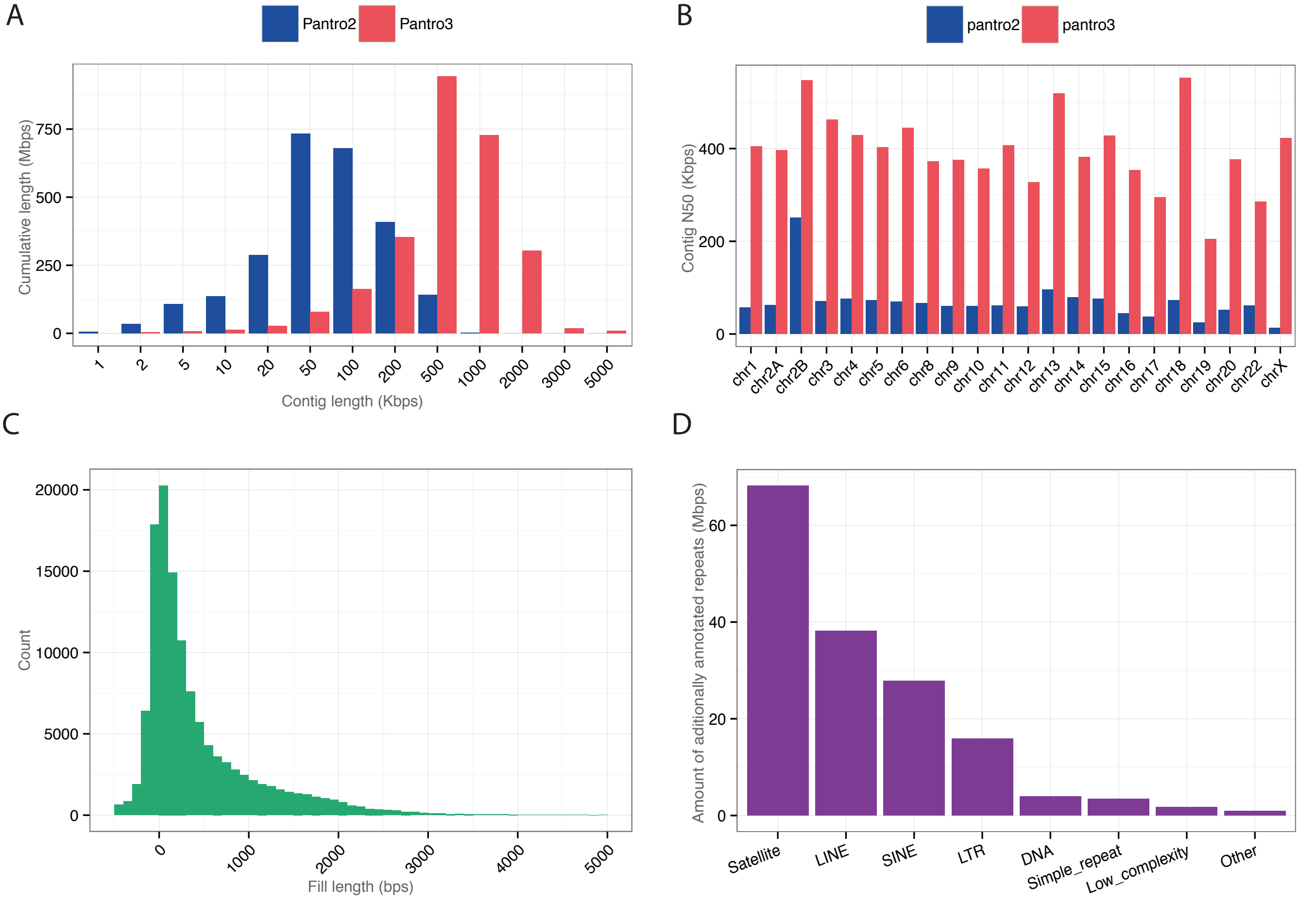

Point by point response to reviewers

Click here to access/download
Supplementary Material
Kuderna_et_al_Point_by_point_response_to_rev.docx

Click here to access/download
Supplementary Material
Kuderna_et_al.SUPPLEMENTARY_resubmission.docx

Report Reviewer 3

Click here to access/download
Supplementary Material
Referee 3_Miller.odt
