# Supplementary material for: A 3-way hybrid approach to generate a new high-quality chimpanzee reference genome (Pan_tro_3.0)
Source: Gigascience. 2017 Oct 30;6(11):1–6. doi: 10.1093/gigascience/gix098 (PMC5714192; doi:10.1093/gigascience/gix098)
Supplement: Supplement materials [file gix098_supp.docx]

S1: Assembly Generation 2

S2: Genome Upgrading and long read comparison 4

S3: Segmental Duplications in Pan_Tro_3.0 7

S4: Gene annotation 8

Supplementary Figures 10

Supplementary Tables 33

References 38

# S1: Assembly Generation

All sequencing data is derived from a cell line from a single western chimpanzeeÍ, available from the Coriell Biorepository under the identifier S006007.

We assembled 4 lanes of 250 bps paired end reads derived from a single library with a fragment size of 400 bps and sequenced on the Illumina HiSeq 2500 Platform using DISCOVAR Denovo (version 51280, default parameters) [1]. These correspond to 242 Gbp of raw sequence data, or 75-fold coverage assuming a genome size of 3.2 Gbp. The data was assembled into 1,594,645 contigs, with a total assembly length of 3,349,198,710 bps and an N50 of 71,979 bps. We introduced a lower bound sequence cutoff of 1 Kbp before scaffolding, discarding 1,478,075 short sequences. Scaffolding was performed with HiRise using one Chicago library produced by Dovetail genomics [2] sequenced on one lane on the Illumina HiSeq 2000 platform. The resulting assembly had 48,621 sequences. This assembly was used as an input for PBJelly (version 14.9.9) [3] in order to fill any remaining gaps with PacBio data sequenced with P5C3 chemistry. PBJelly was run retaining one single best hit per read with a minimum identity of 75%. Base substitutions and small insertion-deletion errors were then corrected with *Raccoon*, a reference assembly base substitution and small indel correction pipeline (available at https://github.com/lukud/raccoon-). Briefly, *Raccoon* maps the raw data used to create the assembly back onto the scaffolds, and performs variant calling of base substitutions and small insertions and deletions (>7bps). These calls are used to create an intermediary assembly, onto which data is mapped back. A decrease in read depth in regions of putative corrections is used as a criterion to discard false corrections. Assembled Pan_tro_3.0 scaffold sequences were then aligned against both Pan_tro_2.1.4 and human GRCh38 references using NUCMER [4] and, after breaking the scaffolds into 1kb segments, aligned using BLAT [5] to aid in defining uniquely aligning segments and precise locations of possible breaks. Further, BLAT was used to align BAC end sequences, fosmid end sequences, along with human (<ftp://hgdownload.cse.ucsc.edu/goldenPath/hg38/bigZips/mrna.fa.gz>) and chimpanzee mRNA sequences (<ftp://hgdownload.cse.ucsc.edu/goldenPath/panTro4/bigZips/mrna.fa.gz>) to the Pan_tro_3.0 scaffold sequences. Where at least two BAC and/or fosmid end sequences suggested possible or where alignments to the human or chimpanzee genome suggested at least 25 Kbp of sequence placed non-contiguously against the human or chimpanzee genome, alignments were manually reviewed and breaks were introduced when warranted. After any breaks, the final scaffolds were again aligned against both Pan_tro_2.1.4 and human GRCh38 references using NUCMER and, after breaking the scaffolds into 1 Kbp segments, aligned using BLAT. Scaffolds were ordered and oriented based on their alignments to the human and chimpanzee genomes with known human/chimpanzee breakpoints included. In the final phase finished BAC clones from the male 'Clint' chimpanzee were integrated into the assembly. Finally, centromeres were placed along each chromosome using centromere localization data from the human genome.

Although Pan_tro_2.1.4 is a whole genome shotgun assembly, clone-by-clone sequencing and assembly has been used to essentially finish the chromosomes 7, 21 and Y. While Clint is the main DNA donor for Pan_tro_2.1.4, there are finished clones from a BAC library (RPCI-43) constructed from a second individual (‘Donald’) that were also incorporated into the assembly. This individual contributed approximately 28% of the sequence of chromosome 7 and virtually all of chromosome 21. As we wished to construct a reference assembly derived from a single individual, we excluded clones from the RPCI-43 library, leading to a decrease in contiguity in contig N50 from 5.03 Mbp to 1.68 on chromosome 7, and from 9.98 Mbp to 433 Kbp on chromosome 21.

Whole genome alignments to Pantro-2.1.4 were produced using last (version 646) keeping best only reciprocal placements.

# S2: Genome Upgrading and long read comparison

Although PacBio and Illumina TruSeq SLR are fundamentally different technologies, both give long-range genomic information that was unprecedented with previous sequencing platforms. In our data, the median read length of PacBio is 6600 Kbp. In some cases (N=25) read lengths exceeded 50 Kbp and go as high as 100,919 bp (> 100 Kbp). However, closer inspection shows that although identifiable as deriving from the target genome, these long reads (>50 Kbp) bare little resemblance and identity with it upon mapping and are thus more likely to be sequencing errors. The read length of the TruSeq SLR data ranges up to 20,828 bps. The median read length is of 1,499 bps including reads with a length below 1.5 Kbp, and 3,219 bps excluding them. This is worth mentioning, as the supplier does not consider reads with a length below 1.5 Kbp to be SLR, and thus labels them differently. For our further analysis, we included all the data available. The read length distribution shows an increased amount of reads around 10 Kbp due to the way libraries for this technology are prepared (Kuleshov et al., 2014).

We mapped both the PacBio and the TruSeq SLR datasets onto Pan_tro_2.1.4 using blasr and retaining only a single best hit per read, as most ambiguous cases of hits due to repeats should be resolvable with this read length [7]. In the TruSeq data, out of 8,244,511,537 sequenced bases, 8,099,022,212 were mappable onto the reference (98.2%). For the PacBio data, out of 28,783,071,317 sequenced bases 20,517,073,482 were mappable onto the reference, or 71.3 %. Out of 3.3 Gbp in the reference assembly, around 2.9 Gbp are covered by at least one read in the PacBio data, or 87%. However, not considering gapped bases, i.e. bases represented by N’s in the assembly, the percentage of the genome that is covered by at least one read increases to 93%. Only around 70% percent of bases in the reference are covered with at least one read on the TruSeq SLR data at 2.7-fold coverage, or 76% not considering gaps. We also produced a down-sampled PacBio dataset that roughly equals the TruSeq SLR data in amount of mapped bases, to see if the observed effects are attributable solely to differences in coverage. In this set, 80% of the reference is covered by at least one read, or 85% not considering gaps. These numbers suggest that non-random processes are responsible for the differences in unmapped bases between PacBio and TruSeq SLR data at equal coverage. Figure S 1 shows the distribution of coverages across the different datasets.

We then sought to identify the capabilities of both datasets to resolve repetitive elements in the genome. To asses this question, we intersected the genomic intervals of that are not covered by each dataset with the repeatmasker annotation from UCSC, and counted the amount of bases falling within each repeat. There are a total of 37.5 Mb, 155.4 Mbp and 312.3 Mbp uncovered within annotated repeats in the full PacBio, the down-sampled PacBio and the TruSeq SLR datasets. The TruSeq SLR data shows a pronounced bias towards not covering repetitive elements. This likely is due to the fact that these reads are not sequenced from single molecules, but are the product of a library preparation protocol that seeks to reduce sequence assembly complexity by separating fragments of around 10 Kbp into different wells, sequencing them with short read technologies and then trying to reconstruct the original fragment. This bias is absent in the PacBio data, even when down sampling to comparable coverage. Figure S 2 shows counts of non-covered bases intersecting repeats. Figure S 3 shows counts of non-covered bases intersecting repeats normalized by the number of bases annotated for the repeat. Note that these numbers are sensible to repeats that make up little in terms of length. Nevertheless, the trend in repeat resolution is the same with or without normalizing, albeit the affected repeats change.

Long reads offer a novel possibility of filling gaps within an assembly that was inaccessible by previous technologies. This is achieved by mapping the data and identifying reads that either sit on boundaries of gaps into or span them. These reads, together with flanking regions, are then extracted and assembled to produce a fill sequence for the gap. We applied PBJelly [3], an automated gap-filling pipeline specifically designed for this purpose, with both datasets using default parameters to get rough estimates of how many gaps are closable with either technology. In this context, a gap was defined as a stretch of at least 25 consecutive N’s in the assembly. Out of 124,401 such gaps within the assembly, 76,841 could be filled with PacBio (~50%), and 30,422 could be filled with TruSeq SLR (~25%). Of the remaining gaps, 6,066 could be shortened with TruSeq and 6,769 with PacBio. We additionally ran a combination of both datasets. Comparing the results of this runs shows that there are comparatively few cases (N=3,347) for which a gap closure could be achieved with TruSeq SLR exclusively and not by PacBio. Figure S 4 gives an overview of closed and shortened gaps by separate by dataset.

# S3: Segmental Duplications in Pan_Tro_3.0

We applied two *in silico* methods to discover segmental duplications in the Pan_tro_3.0 assembly. Whole-genome assembly comparison (WGAC) compares repeat-free sequence of the assembly to itself to identify duplicated sequences greater than or equal to 1 Kbp and with 90% identity or higher [8]. Whole-genome shotgun sequence detection (WSSD) aligns whole-genome shotgun (WGS) reads to the assembly and identifies large, highly identical regions (≥10 Kbp, >94% identity) with a higher read depth compared to known unique regions [9]. We mapped 31,366,275 Sanger WGS reads from one male chimpanzee to panTro3 and called duplications in 5 Kbp windows with a read depth >107 (threshold determined by mean read depth in unique regions plus three standard deviations where mean=53.3, std dev=17.1), 200 bps of unmasked sequence, and 200 bps of sequence with a Phred quality >30.

In the assembly-based analysis (WGAC) of Pan_tro_3.0, we discovered 178,245 redundant pairwise alignments corresponding to 388 Mbp of non-redundant sequence (12.39% of the genome sequence excluding gaps) (Table S 1). The majority of these (321 Mbp) were inter-contig duplications, with 120 Mbp being within contigs. Excluding unplaced contigs, we found 140 Mbp of non-redundant duplicated sequence in Pan_tro_3.0 (both within and between) chromosomes (4.46% of the non-gap bases in the genome), which is more than the 110 Mbp of a previous chimpanzee reference (panTro2) and closer to the 170 Mbp recent human reference (GRCh38) (Table S 1). WSSD shows only 63 Mbp of duplicated sequence (compared to 284 Mbp WGAC ≥10 Kbp, >94% identity) with 51 Mbp assigned to chromosomes (compared to 68 Mbp WGAC ≥10 Kbp, >94% identity).

# S4: Gene annotation

Two sets of whole genome alignments between human assembly hg38 and both Pan_tro_2.1.4 and Pan_tro_3.0 were generated using progressiveCactus[10]. The first set used the common reference assemblies for great apes, and the second set used updated assemblies where possible. Both alignments used Rhesus macaque (rheMac8), squirrel monkey (saiBoli1), bushbaby (otoGar3), and mouse (mm10) as outgroups. Using these alignments, transcripts from Gencode annotation set V24 (available at https://www.gencodegenes.org/) were lifted over from human to both chimpanzee assemblies using transMap[11]. These comparative transcripts were given to Augustus as strong hints along with additional RNAseq information in a per-locus fashion to try and fix small gaps or unaligned exons.

Both the original transMap and the Augustus derivative transcripts were evaluated by a collection of binary classifiers. These classifiers diagnose differences such as frameshifts, coding indels, invalid splice sites, incomplete alignment, and unknown base content. Based on these classifiers, each transcript was categorized as one of excellent, pass or fail. Based on their categorization and fidelity to the reference, a consensus was produced between these two annotation sets. In this consensus, all transcripts are represented if they are at least passing. If all transcripts for a gene fail, then one longest representative for the locus is picked.

This process highlights improvements in the new assembly. In total, 20,373 coding genes mapped over to Pan_tro_3.0 compared to 20,165 for Pan_tro_2.1.4. Notably, the number of transcripts with frame-shifting indels decreased from 11,280 to 4,743. This suggests that there were small assembly errors in Pan_tro_2.1.4 that have been corrected in Pan_tro_3.0. The number of transcripts with paralogous mappings increased from 4.938 to 5.265 in Pan_tro_3.0 due to Pan_tro_3.0 being a more comprehensive assembly with fewer collapsed gene families and segmentally duplicated regions. Table S 3 lists a comparison of annotation statistics between Pan_tro_2.1.4 and Pan_tro_3.0 for annotations performed in the same way.

# Supplementary Figures

Figure 1

Flowchart of assembly process


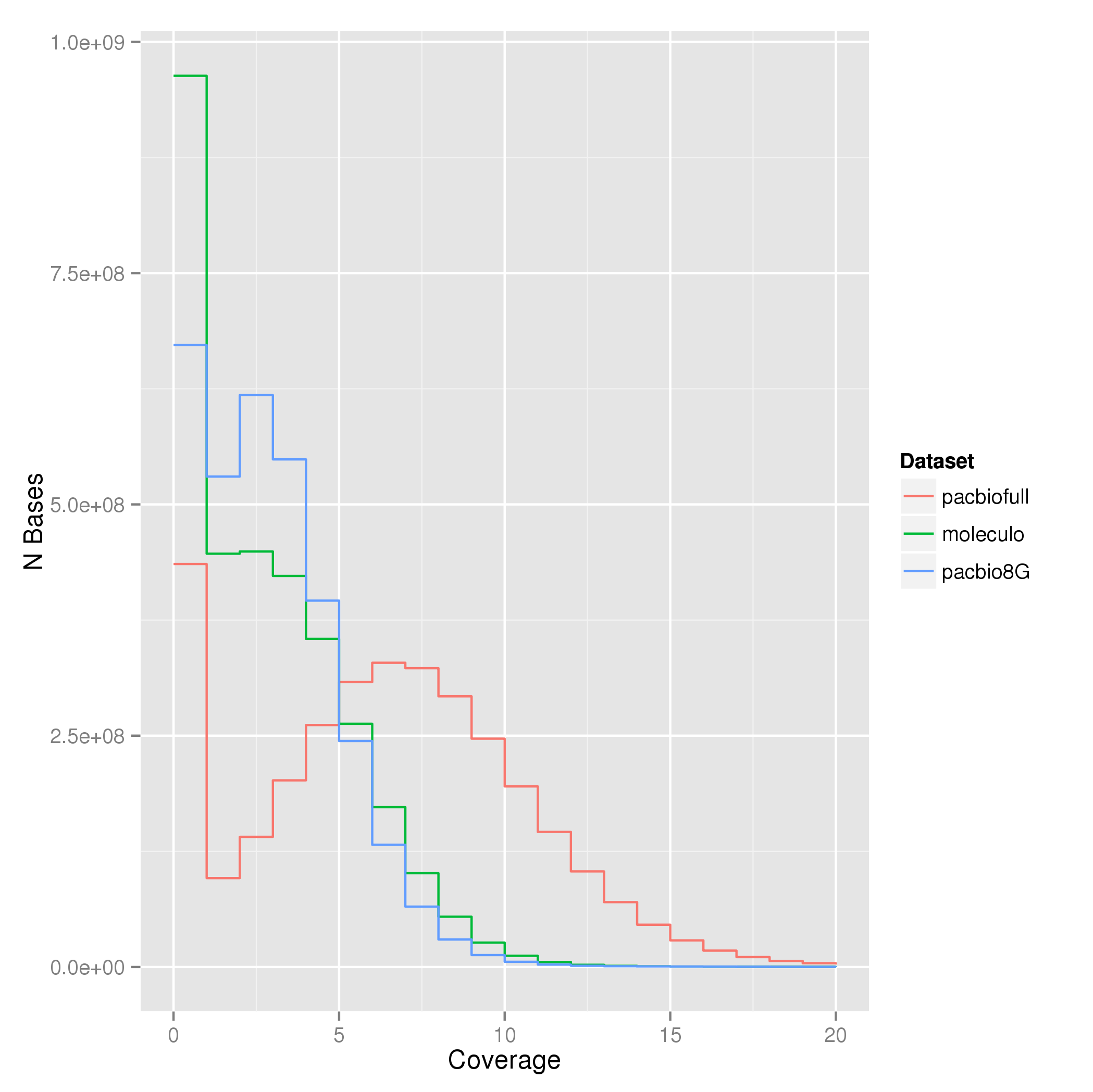


Figure S 1

Coverage Vs number of bases over Pantro-2.1.4. The green line shows the TruSeq SLR data (=Moleculo), the red line the PacBio data. The blue line shows a downsampled PacBio data that is equivalent to the TruSeq SLR data in amount of mapped bases. The main focus of interest lies on the number of bases that are not covered by a single read across the different datasets. At equal mapping coverage (~8 Gbp) TruSeq SLR shows around 1.5x as many bases without a single read mapping them. Gaps are not excluded from the analysis.

Figure S 2

Counts of bases that are not covered by any single read intersecting with repeats. A clear trend towards incorporating more repeats in the TruSeq SLR data can be seen.

Figure S 3

Counts of bases that are not covered by any single read intersecting with repeats normalized by total number of bases annotated within each repeat


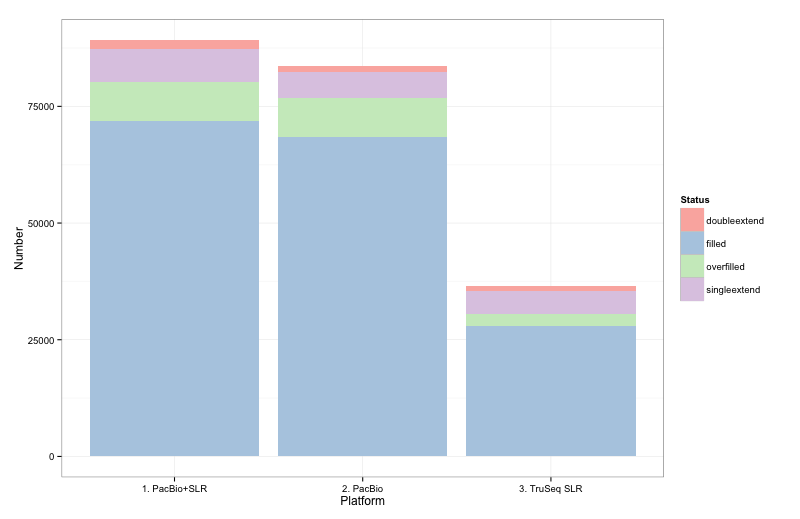


Figure S 4

Status of gaps after running PBJelly with the PacBio and the TruSeq SLR data, as well as a combination of both. Overfilled gaps are closed gaps with fill sequences longer than the predicted gaps size. Single- and double extend are gaps that were shortened from one or both sides. There are relatively few gaps that are addressed by a combination of both PacBio and TruSeq SLR and not PacBio alone.


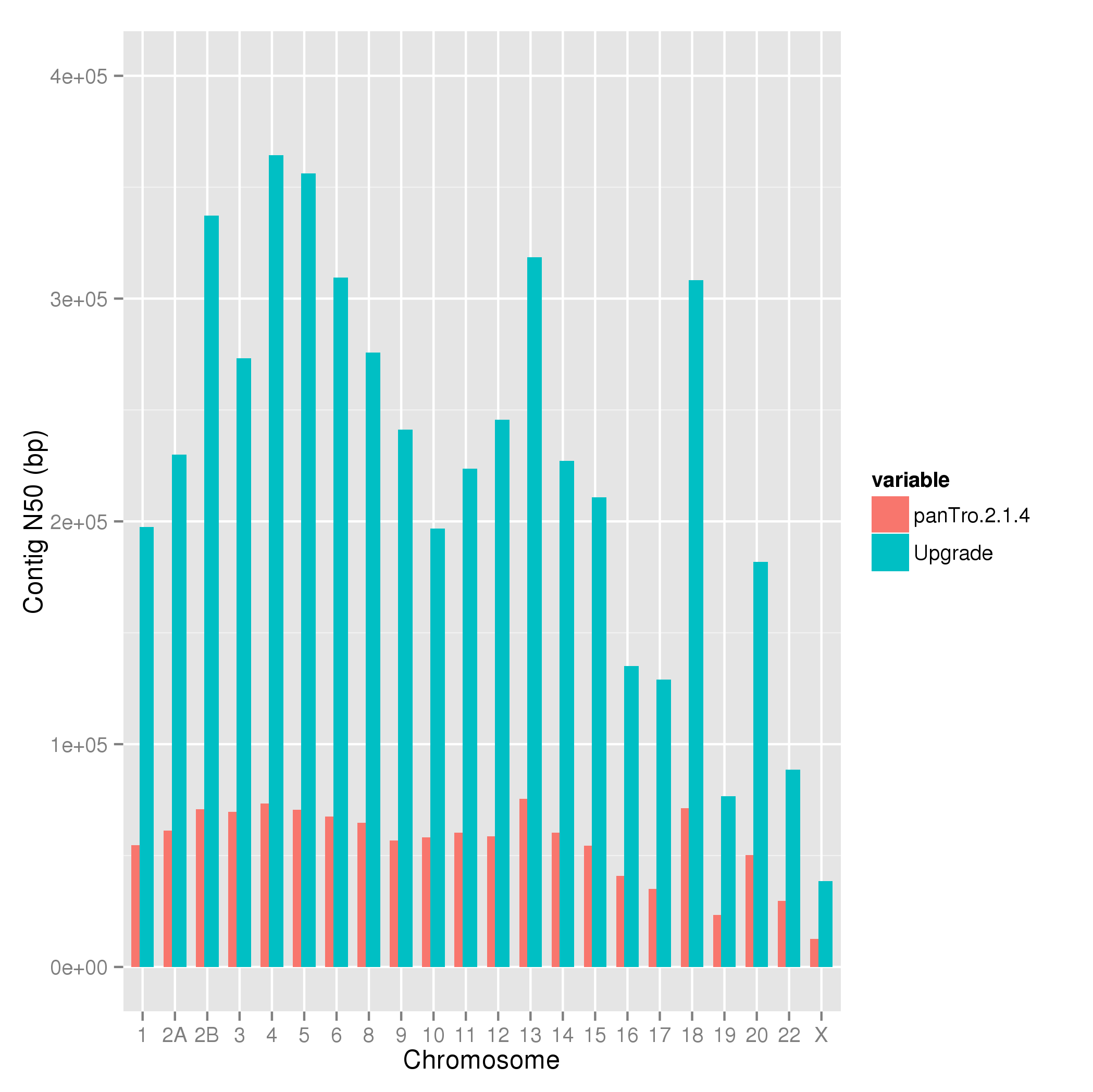


Figure S 5

Increase of contig N50 across all non-finished chromosomes after filling gaps with PacBio data. Approximately 4-fold increase in continuity can be seen across all assessed chromosomes.


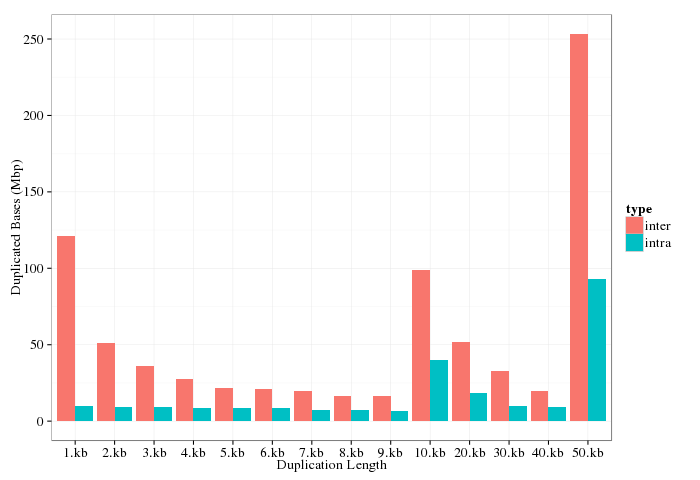


Figure S 6

Length Distribution of Segmental Duplications on all Pan_tro_3.0 Contigs, 1-50 Kbp


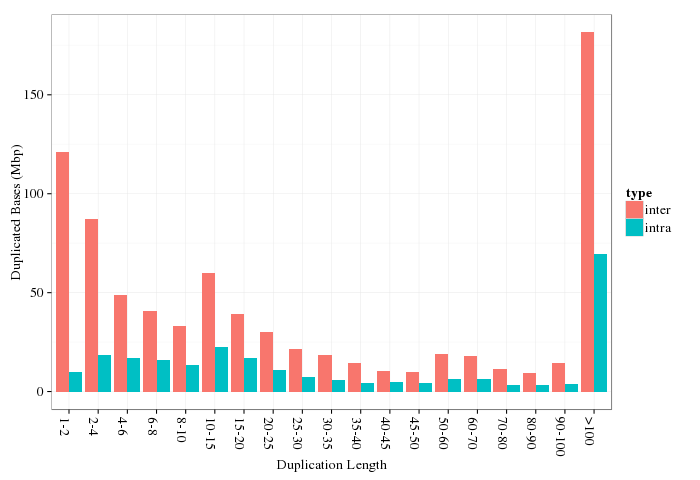


Figure S 7

Length Distribution of Segmental Duplications on all Pan_tro_3.0 Contigs, 1-100 Kbp


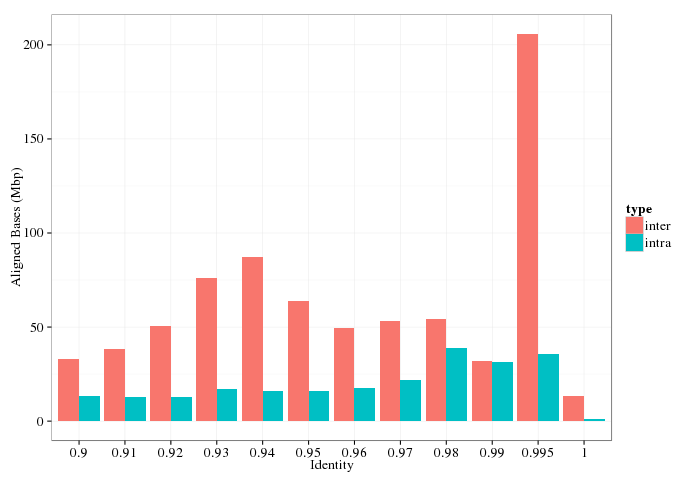


Figure S 8

Similarity Distribution of Segmental Duplications in All Pan_tro_3.0 Contigs


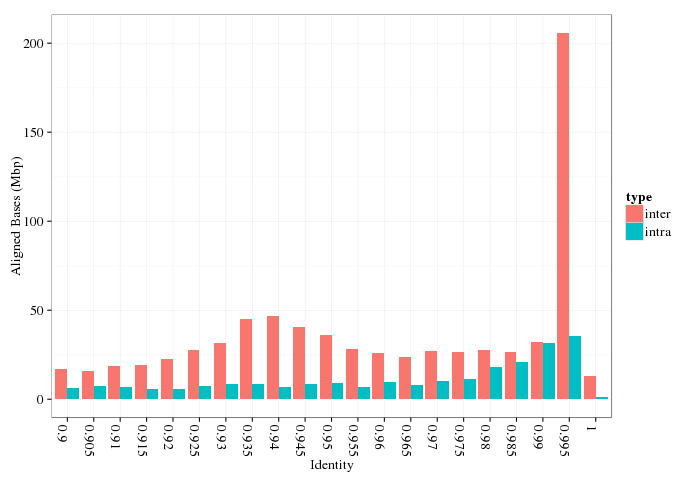


Figure S 9

Similarity Distribution of Segmental Duplications of All Pan_tro_3.0 Contigs (finer resolution)


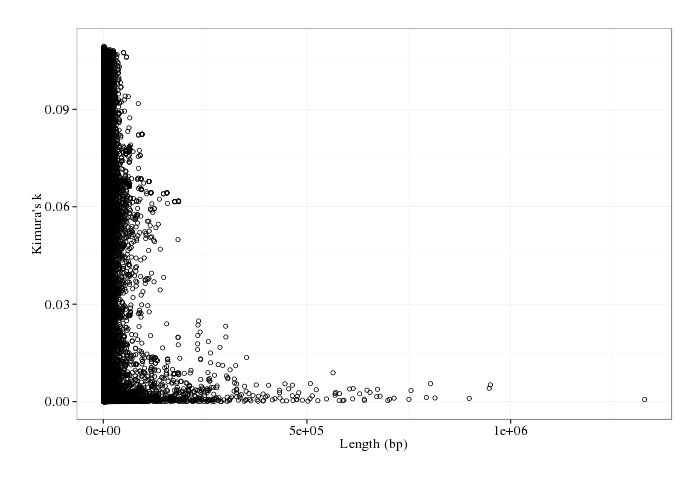


Figure S 10

Kimura’s K vs Length for All Contigs of Pan_tro_3.0


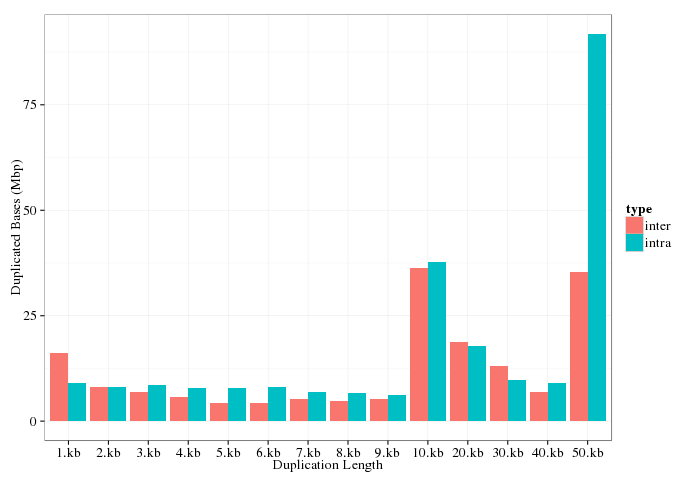


Figure S 11

Length Distribution of Segmental Duplications on Pan_tro_3.0 chromosomal scaffolds, 1-50 Kbps


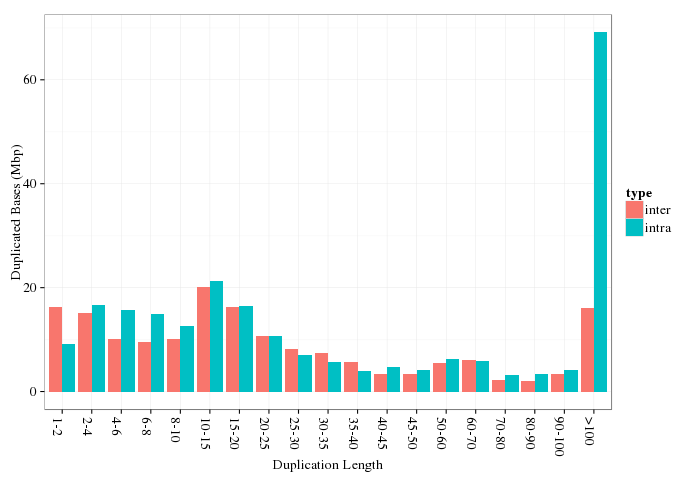


Figure S 12

Length Distribution of Segmental Duplications on Pan_tro_3.0 chromosomal scaffolds, 1-100 Kbps


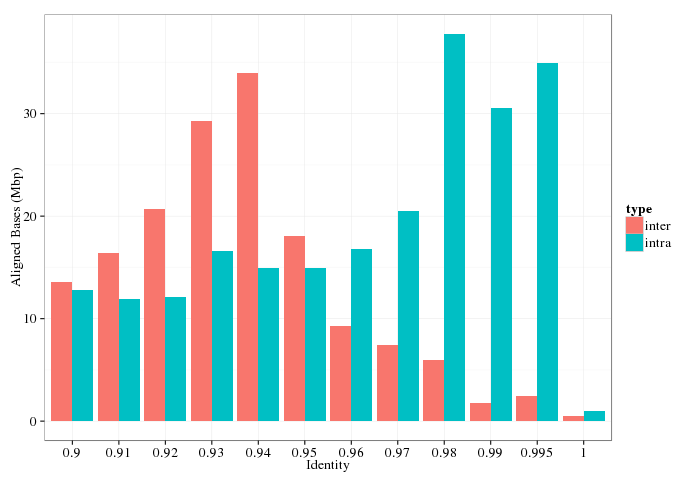


Figure S 13

Similarity Distribution of Segmental Duplications in chromosomal scaffolds of Pan_tro_3.0


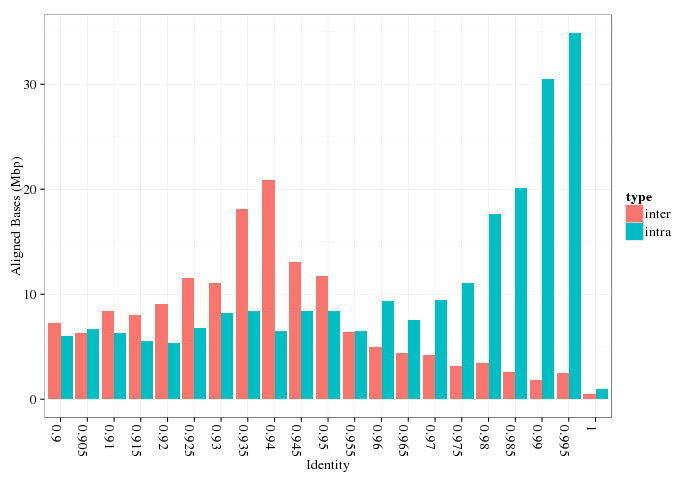


Figure S 14

Similarity Distribution of Segmental Duplications in chromosomal scaffolds of Pan_tro_3.0 (finer resolution)


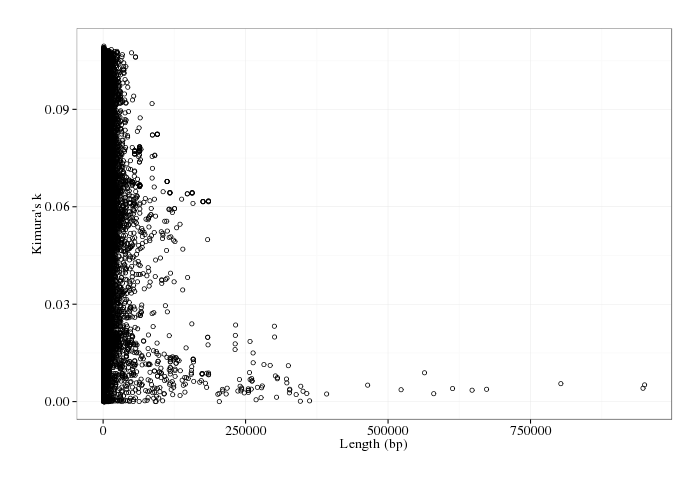


Figure S 15

Kimura vs Length in chromosomal scaffolds of Pan_tro_3.0


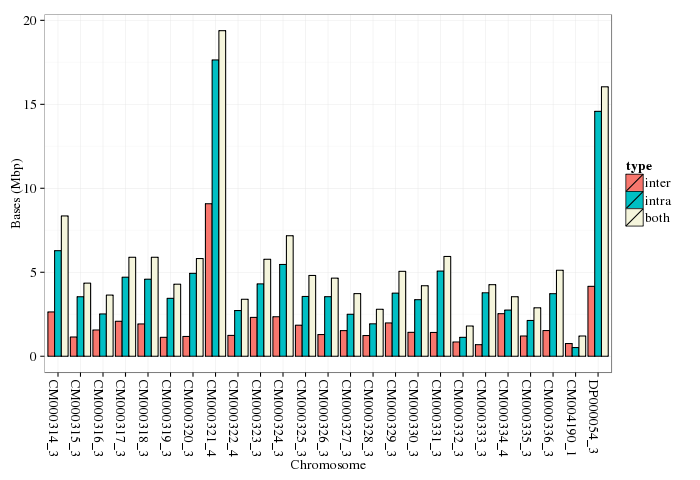


Figure S 16

Nonredundant Segmental Duplication Bases Between Chromosomes (no unplaced contigs). CH000321_4 corresponds to human chromosome 7 and DP000054.3 corresponds to human chromosome Y.


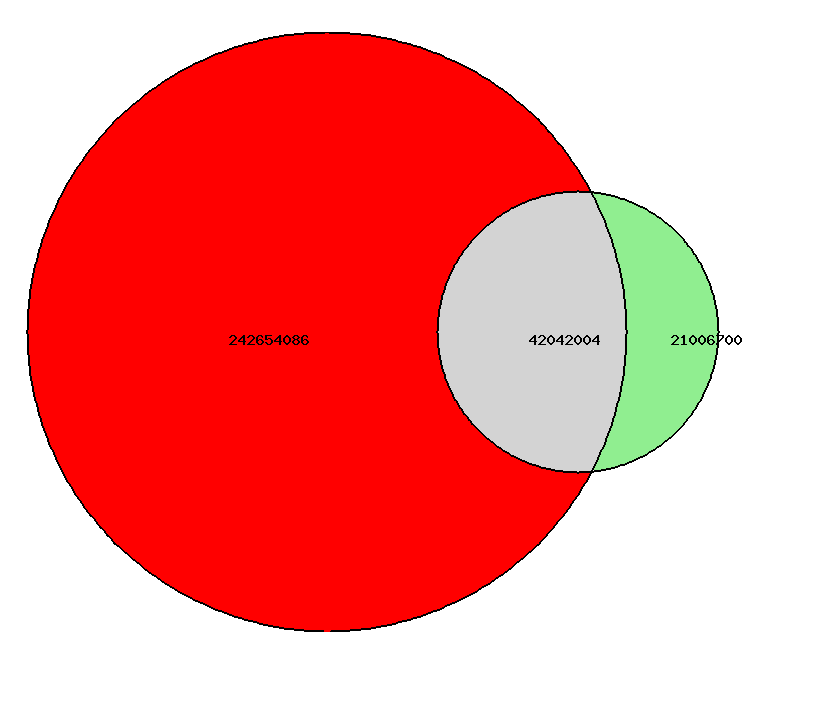


Figure S 17

Segmental Duplication Bases: genome wide WGAC vs WSSD


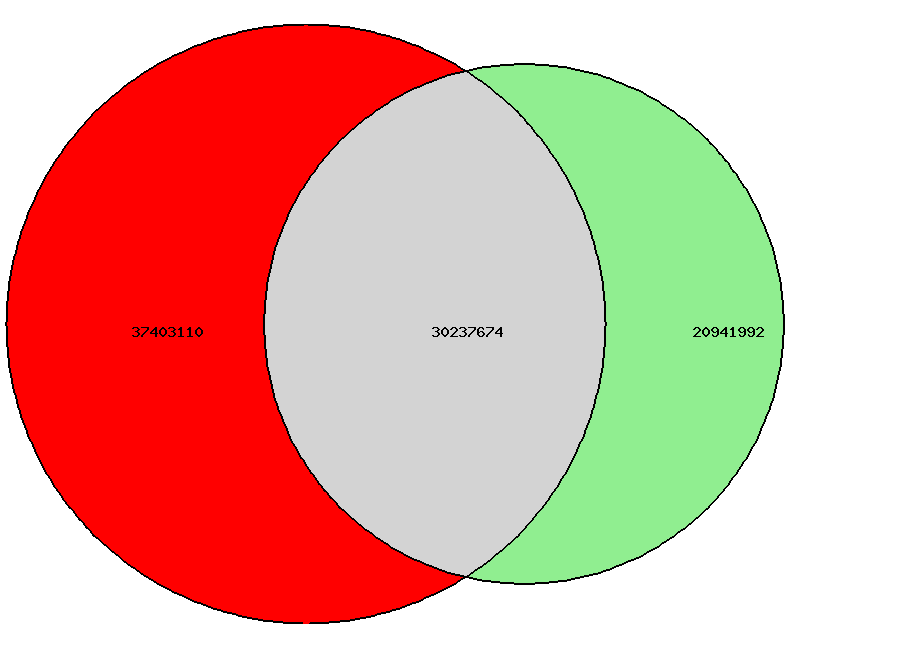


Figure S 18

Segmental Duplication Bases on chromosomal scaffolds: WGAC vs WSSD

Figure S 19

Gap length distribution of unfilled gaps in Pantro_3.0, with a detectable counter-part in Pan_tro_2.1.4

Figure S 20

Gap length distribution of gaps in Pantro_3

Figure S 21

Comparison of repeat content between Pantro_2.1.4 and Pan_tro_3

Figure S 22

Repeat content for lower abundance repeats poorly visible in Figure S21

# Supplementary Tables

| **Category** |  |  | Pan_tro_3.0 | | panTro2 | GRCh38 |
| --- | --- | --- | --- | --- | --- | --- |
| Total genome length | |  | 3.231.154.112 | | 3.350.396.789 | 3.209.286.105 |
| Chrom length (genome without gaps) | | | 3.132.603.062 | | 2.909.223.816 | 3.049.315.783 |
| Number of WGAC pairs | |  | 178.245 | | 192.546 | 69.906 |
| Number of inter contig | |  | 153.382 | | 106.353 | 47.878 |
| Number of intra contig | |  | 24.863 | | 86.193 | 22.028 |
| Total nonredundant bases | |  | 388.403.898 | | 221.999.093 | 175.457.472 |
| Inter-contig nonredundant bases | |  | 320.578.653 | | 148.358.084 | 91.305.737 |
| Intra-contig nonredundant bases | |  | 120.078.319 | | 113.501.320 | 125.039.858 |
| Just >=10kb and > 94% identity (like wssd) | | | 284.696.090 | | 23.929.056 | 173.799.876 |
| **Just chromosomes (no random, unplaced, or small contigs)** | | | | |  |  |
| Nonredundant bases | |  | | 140.015.190 | 109.943.135 | 169.583.314 |
| above as % of genome without gaps | | | | 4,46 | 3,77 | 5,56 |
| Inter-chromosome nonredundant bases | | | | 49.088.087 | 34.169.144 | 83.664.117 |
| Intra-chromosome nonredundant bases | | | | 112.518.129 | 84.317.740 | 123.959.688 |
| Just >=10 Kbp and > 94% identity (like wssd) | | | | 67.640.784 | 13.667.108 | 165.216.961 |
| **WSSD** |  |  | |  |  |  |
| Bases (all contigs) | |  | | 63.048.704 | 149.402.118 | 192.337.115 |
| Bases (just chromosomes) | |  | | 51.179.666 | 67.277.418 | 183.908.509 |
| Counts (all contigs) | |  | | 3.257 | 36.146 | 2.246 |
| Counts (just chromosomes) | |  | | 2.325 | 8.143 | 2.144 |

Table S 1

Overview of segmental duplication content for Pan_tro_3.0, Pan_tro2 and GRCh38

| **Assembly** | **contig_N50** |
| --- | --- |
| Pan_tro 3.0 | 384816 |
| P_pygmaeus_2.0.2 | 15648 |
| gorGor4 | 52934 |
| DauMad_1.0 | 3358 |
| Charlie1.0 | 13295 |
| Emacaco_refEf_BWA_oneround | 20038 |
| Eflavifronsk33QCA | 27331 |
| Cebus_imitator-1.0 | 41196 |
| Callithrix jacchus-3.2 | 29273 |
| Nleu_3.0 | 35148 |
| Tarsius_syrichta-2.0.1 | 38165 |
| Mmur_2.0 | 182929 |
| OtoGar3 | 27100 |
| SaiBol1.0 | 38823 |
| panpan1.1 | 66676 |
| Panu_2.0 | 40262 |
| Macaca_fascicularis_5.0 | 86040 |
| Chlorocebus_sabeus 1.1 | 90449 |
| Rrox_v1 | 77151 |
| Mmul_8.0.1 | 107156 |
| Cang.pa_1.0 | 38363 |
| Mleu.le_1.0 | 31346 |
| Anan_1.0 | 28503 |
| Caty_1.0 | 112942 |
| Mnem_1.0 | 106897 |
| Pcoq_1.0 | 28129 |

Table S 2

Contig N50 of all primate assemblies in the RefSeq category ‘representative’ (July 2016)

| (V24 has 21920 coding genes, 86909 coding transcripts) | Pan_tro_2.14 | Pan_tro_3.0 |
| --- | --- | --- |
| Coding genes | 20165 | 20373 |
| Coding transcripts | 75499 | 77858 |
| Average coverage | 96.87% | 98.86% |
| Average identity | 99.08% | 99.12% |
| # of transcripts with multiple mapping | 4938 | 5265 |
| # of transcripts which may be broken by scaffold gap | 25 | 32 |
| # of transcripts which have frame shifting indels | 11280 | 4743 |
| # of transcripts which have frame shifting indels which survived consensus finding | 8421 | 3382 |
| # of transcripts with 100% coverage | 45836 | 54360 |

Table S 3

Summary statistics for the gene annotation

| **Repeat Type** | **Pan_tro_2.1.4 bases** | **Pan_tro_2.1.4 counts** | **Pan_tro_3.0 bases** | **Pan_tro_3.0 counts** |
| --- | --- | --- | --- | --- |
| DNA | 241746 | 1971 | 249241 | 2024 |
| DNA/MULE-MuDR | 602519 | 1795 | 642123 | 1887 |
| DNA/Merlin | 16989 | 61 | 15967 | 53 |
| DNA/MuDR | 107731 | 253 | 108602 | 253 |
| DNA/PiggyBac | 502174 | 2180 | 526424 | 2293 |
| DNA/PiggyBac? | 45858 | 241 | 48228 | 252 |
| DNA/TcMar | 34065 | 196 | 36190 | 210 |
| DNA/TcMar-Mariner | 2842902 | 16408 | 2975136 | 17327 |
| DNA/TcMar-Pogo | 4600 | 41 | 4393 | 39 |
| DNA/TcMar-Tc2 | 1680251 | 8115 | 1744608 | 8478 |
| DNA/TcMar-Tigger | 36163097 | 116254 | 37603654 | 120775 |
| DNA/TcMar? | 248341 | 1377 | 255256 | 1421 |
| DNA/hAT | 1200505 | 8926 | 1244538 | 9242 |
| DNA/hAT-Blackjack | 3513060 | 20672 | 3651680 | 21408 |
| DNA/hAT-Charlie | 46713552 | 262415 | 48448595 | 272451 |
| DNA/hAT-Tip100 | 8595512 | 45273 | 8932465 | 47221 |
| DNA/hAT-Tip100? | 303020 | 2166 | 317977 | 2276 |
| DNA/hAT? | 138671 | 582 | 135906 | 558 |
| DNA? | 304201 | 2120 | 312918 | 2186 |
| LINE/CR1 | 11043898 | 62066 | 11453643 | 64287 |
| LINE/Dong-R4 | 114284 | 541 | 120016 | 571 |
| LINE/L1 | 518039575 | 1013946 | 550474469 | 1058737 |
| LINE/L2 | 97907278 | 458790 | 103046650 | 477861 |
| LINE/Penelope | 11874 | 58 | 12014 | 59 |
| LINE/RTE-BovB | 69395 | 634 | 72639 | 659 |
| LINE/RTE-X | 3433923 | 15443 | 3532026 | 15887 |
| LTR | 729842 | 3338 | 758891 | 3463 |
| LTR/ERV1 | 82167086 | 180004 | 89894701 | 190163 |
| LTR/ERV1? | 269084 | 1492 | 279030 | 1550 |
| LTR/ERVK | 8986530 | 11267 | 9387033 | 11632 |
| LTR/ERVL | 58533216 | 164447 | 60939562 | 170285 |
| LTR/ERVL-MaLR | 111246214 | 351262 | 116345904 | 365300 |
| LTR/ERVL? | 571643 | 2224 | 601081 | 2340 |
| LTR/Gypsy | 3020026 | 14092 | 3087673 | 14450 |
| LTR/Gypsy? | 1472961 | 7887 | 1528974 | 8171 |
| LTR? | 1229579 | 5823 | 1263614 | 6002 |
| Low_complexity | 17051672 | 389178 | 18825295 | 411656 |
| RC/Helitron | 395982 | 1820 | 411802 | 1911 |
| RC?/Helitron? | 65584 | 433 | 67365 | 441 |
| RNA | 129740 | 797 | 131948 | 811 |
| Retroposon | 3259356 | 4171 | 4154395 | 5185 |
| SINE/Alu | 302267032 | 1184240 | 327004037 | 1267877 |
| SINE/Deu | 316687 | 3048 | 327744 | 3151 |
| SINE/MIR | 81914036 | 586805 | 84896359 | 608669 |
| SINE/tRNA | 509230 | 3980 | 531550 | 4168 |
| SINE? | 52350 | 475 | 54279 | 493 |
| Satellite | 6136819 | 13435 | 14267292 | 25539 |
| Satellite/Y-chromosome | 21749175 | 6318 | 21222379 | 5404 |
| Satellite/acro | 67810 | 122 | 61132 | 132 |
| Satellite/centr | 1966430 | 5534 | 1877856 | 4232 |
| Satellite/telo | 4931106 | 2402 | 65542641 | 40487 |
| Simple_repeat | 27724076 | 440134 | 31196727 | 473613 |
| Unknown | 427226 | 2753 | 437498 | 2801 |
| rRNA | 227327 | 1838 | 246808 | 1929 |
| scRNA | 128159 | 1352 | 137720 | 1454 |
| snRNA | 339006 | 4349 | 356138 | 4559 |
| srpRNA | 281765 | 1764 | 299955 | 1851 |
| tRNA | 110826 | 1929 | 115112 | 2009 |

Table S 4

Repeat content for Pan_tro_2.1.4 and Pan_tro_3.0

# References

1. Weisenfeld NI, Yin S, Sharpe T, Lau B, Hegarty R, Holmes L, et al. Comprehensive variation discovery in single human genomes. Nat. Genet.; 2014;1–8.

2. Putnam NH, Connell BO, Stites JC, Rice BJ, Hartley PD, Sugnet CW, et al. Chromosome-scale shotgun assembly using an in vitro method for long-range linkage. Genome Res. 2016;1–25.

3. English AC, Richards S, Han Y, Wang M, Vee V, Qu J, et al. Mind the gap: upgrading genomes with Pacific Biosciences RS long-read sequencing technology. PLoS One Public Library of Science; 2012;7:e47768.

4. Delcher AL, Kasif S, Fleischmann RD, Peterson J, White O, Salzberg SL. Alignment of whole genomes. Nucleic Acids Res. 1999;27:2369–76.

5. Kent WJ. BLAT — The BLAST -Like Alignment Tool. Genome Res. 2002;12:656–64.

6. Kuleshov V, Xie D, Chen R, Pushkarev D, Ma Z, Blauwkamp T, et al. Whole-genome haplotyping using long reads and statistical methods. Nat. Biotechnol; 2014;32:261–6.

7. Chaisson MJP, Huddleston J, Dennis MY, Sudmant PH, Malig M, Hormozdiari F, et al. Resolving the complexity of the human genome using single-molecule sequencing. Nature2014

8. Bailey J a, Yavor AM, Massa HF, Trask BJ, Eichler EE. Segmental Duplications : Organization and Impact Within the Current Human Genome Project Assembly Segmental Duplications : Organization and Impact Within the Current Human Genome Project Assembly. Genome Res. 2001;11:1005–17.

9. Bailey J a, Gu Z, Clark R a, Reinert K, Samonte R V, Schwartz S, et al. Recent segmental duplications in the human genome. Science. 2002;297:1003–7.

10. Paten B, Earl D, Nguyen N. Cactus : Algorithms for genome multiple sequence alignment Cactus : algorithms for genome multiple sequence alignment. 2011;1512–28.

11. Stanke M, Diekhans M, Baertsch R, Haussler D. Using native and syntenically mapped cDNA alignments to improve de novo gene finding. Bioinformatics. 2008;24:637–44.
