# Supplementary material for: A 3-way hybrid approach to generate a new high-quality chimpanzee reference genome (Pan_tro_3.0)
Source: Gigascience. 2017 Oct 30;6(11):1–6. doi: 10.1093/gigascience/gix098 (PMC5714192; doi:10.1093/gigascience/gix098)
Supplement: Response-to-Reviewer-Comments_Original-Submission.pdf [file gix098_response-to-reviewer-comments_original-submission.pdf]

Author's Response To Reviewer Comments

Dear Dr. Hans Zauner,

Please find attached the resubmission of our revised manuscript GIGA-D-16-00140; A 3-way hybrid approach to generate a new high quality chimpanzee reference genome (Pan_tro_3.0), together with a point by point response of the comments of all reviewers. We believe that we satisfactorily address all the comments in there.

We also wanted to report an issue that has been brought to us after a revision on the assembly. We have detected several small inversions affecting either full contigs within a scaffold, or the end of a contig. Because these events are predominantly rather small, they have previously escaped our notice when assessing (large scale) structural errors using clones, as they are only detectable by fine scale comparisons to the previous chimpanzee assembly, as well as the human genome assembly. Nevertheless, in the spirit of full disclosure, we did not want to resubmit our revised manuscript without addressing the issue first, now included in the final version of the assembly. Altogether, this affects 2,990 fully inverted contigs, amounting for 20.5 Mb of sequence. These were flipped and left in place in a new version of the assembly. Furthermore, there are 1,505 pieces of contigs, where the breakpoint of the inversion lays within the contigs, amounting for 11.1 Mb of sequence. In these cases, we manually went through the flips and decided to move some off to the unplaced portion of the assembly, and inverted some in place, depending on how clearly we could assign them to belonging to the chromosome in question. Cumulatively, there are now 31.6Mb of sequence in the assembly were we have changed the orientation. This affects 1,938 of coding exons amounting for 276,641 bp of coding sequence. Please note that the overall sequence content of the assembly has remained the same.

We are currently in the process of resubmitting our revised assembly to NCBI. For the paper, we have chosen to explicitly state which version of the NCBIs accessions system is being used.

We are looking forward to your reply.

Kind Regards,

Lukas Kuderna & Tomas Marques-Bonet

########

#Please note we are attaching a formatted document for the reviews with all graphics in place. Below you will find the copied text only from that document.

Point by point rebuttal:

Reviewer 1:

Reviewer #1: The authors present several large new datasets of chimpanzee genome sequencing data, and they combine these datasets into a novel, high-quality genome assembly of Pan troglodytes. As the authors state, this is a valuable addition to the set of available genome sequence resources and a vast improvement in genome quality over the existing Pan troglodytes assembly. The manuscript needs some editing and cleaning up, but overall I believe it represents a significant contribution to the field and should eventually be published.

In the "Data description" section, the paper gives an overview of the datasets the authors used. This section would benefit from a clear introduction and description of the sequencing strategies they employed to process these datasets. I suggest that a new figure, in the form of a simple flowchart, could be a helpful visual aid: it would describe the assembly methods that were used to combine the various types of sequencing libraries, and it would illustrate the process of creating the 3-way hybrid intermediary assembly as well as the final (3.0) assembly. Additionally, the "Data description" section is mostly devoid of citations. More citations should be added in order to give proper attribution to the developers of the assembly methods, and to enable the reader to seek more information.

We have sought to clarify on the different sequencing strategies, and have added references where adequate in this section (Goodwin S et al. ,2016; Kuleshov et al, 2014; Putnam et al, 2016). We have also added the flowchart of the assembly process in supplementary figure 1 (see below). Nevertheless, we have only slightly modified the introductory section, as it is our understanding that this is adequate for the data note format.

The authors discuss the sequence content they have added to the chimpanzee genome. It's interesting to see the length distribution of the gaps they have filled (Figure 1C), and I would be curious to see comparative length distributions for gaps they failed to fill, or for gaps they added.

We have added plots of length distributions for both of these cases. Supplementary figure S19 (first figure below) shows the length distribution of gaps we can identify as corresponding between Pantro_2.1.4 and Pan_tro_3, but fail to fill. Supplementary Figure S20 (second figure below) shows the length distribution of gaps present within Pan_tro_3.0. We note, that the overall shape of the distribution is similar, with peaks at small gap sizes.

The detail on the repeat resolution is also fascinating. I think the authors sell themselves short by noting that the repeat fraction of the assembly increases from 50.9% to 52.2%: given that they only increase the assembly sequence length by ~8%, this actually shows that most of the sequence they've added is repeat sequence, which is a useful indicator of the new assembly's added value. Similarly, Figure 1D, which shows the quantities of added repeat sequence for

various repeat types, would be stronger if it also showed the quantities of already-existing repeat sequence for each type.

We have added a plot showing the full comparative repeat content of both Pan_tro2.1.4 and Pan_tro3 at supplementary Figure S21 (first figure below) , as well as a scaled version for repeat families with fewer annotations at Figure S22 (second figure below).

The authors compare the new (3.0) genome assembly to the existing (2.1.4) assembly. They observe a 99.9% overall sequence similarity and note that the 0.1% differences could be explained by SNPs; it would be interesting to see a deeper analysis of these SNPs, although this may be outside the scope of the manuscript.

While we agree that this would be an interesting exploration, we believe, in accordance with the reviewer, that an analysis of these SNPs is out of the scope of this manuscripts' format, especially considering its submission in the form of a GigaScience Data Note.

Also, in the section "Gene annotation", they note a large number of genes with frameshift mutations between the 2.1.4 assembly and the human genome assembly. This is striking, but a fully fair comparison would also mention the number of genes that also contain frameshift mutations (perhaps newly added frameshift mutations) in the 3.0 assembly.

We have now also included the count of frame-shift mutations specific for Pantro_3 for a fully fair comparison in this section (674). We have furthermore clarified, that these frame shifts are not necessarily due to sequence errors in Pan_tro_2.1.4, but might also constitute allelic variation, as the assembly only randomly captures one of two alleles at a given locus.

The conclusion is strong, but it would be stronger with some additional context that describes the achievement in this manuscript. The genome assembly is higher quality. But is it also more efficient, or more economic? Have the authors innovated any new genome assembly methods? Have they demonstrated a technique that could be easily applied to other genome assemblies?

We clarify that our approach should easily be applicable to genomes of similar complexity. Nevertheless, we would like to refrain to comment on efficiency or economical value of the assembly for two reasons: First, the price for sequencing on several platforms has been shown to be extremely dependent on the time of sequence production, and has even dramatically decreased within the timeframe of this manuscript. Second, efficiency in the context of genome assembly is a rather subjective issue, as it not straightforward to decide what to measure efficiency against.

Minor errors:

Section "Assembly generation": "These reads are derived from a 400 bps library, resulting in pairs that overlap over a ~50 bps region". If a 400-bp fragment is sequenced to 250 bp from both ends, wouldn't that result in an overlap of ~100 bp rather than ~50 bp?

We thank the reviewer for catching this error, it is now corrected. The library was size selected to around 450bp, resulting in an overlap of around 50 bp when sequencing 250bp on each side.

Section "Assembly generation": "we observed superior connectivity". The word "connectivity" is unclear in this context; it might be better to simply repeat "contiguity".

We have clarified this sentence by rephrasing it.

Section "Repeat resolution": "We furthermore added 38.2 Mbp of LINE to the assembly, corresponding to over 44,791 additional copies of L1 elements." First of all, this should say "LINEs" rather than "LINE". Secondly, these numbers do not add up. A typical L1 element is 6 Kbp in length; thus, 44,791 copies of L1 elements should necessarily occupy over 260 Mbp of sequence.

We have corrected this flaw by leaving out that number. We erroneously counting L1 annotations, that do not necessarily correspond to fully resolved copies of L1 elements, as repeatmasker annotates partial matches of repetitive elements.

Section "Resolution of segmental duplications": This section should contain more citations, especially for the WGAC and WSSD methods, which are named but not described at all.

We have added references to both these methods in the corresponding section (Bailey et al. 2001; Bailey et al 2002).

Reviewer #2:

The genome of a chimpanzee is an important asset for the study of human evolution, and an high quality reference assembly is long overdue. The authors were able to significantly improve on the previous assemblies. I have no problem with the 3-way hybrid approach they have taken. The paper is written very clearly, and is fully appropriate for the data note format. My recommendation is that this manuscript is accepted without reservation.

We would like to thank the reviewer for the positive feedback.

Reviewer 3:

1. Lines 191 to 212. Is this an exploration of segmental duplications in the genome or over-representation and under-representation in the assembly? What is the support for the conclusion that "segmental duplications are well resolved." The statement of the conclusion is very confusing: "we are likely to be overestimating ... by including an elevated rate of false positive paralogous regions…"

In this section, we explore the representation of segmental duplications within our assembly. We sought to clarify the confusion by rephrasing the concluding sentences of the paragraph to the following:

We then compared Pant_tro_3.0 to the human reference genome assembly GRCh38, an assembly that is based on a BAC hierarchical shotgun assembly strategy and may therefore be considered of gold standard with respect to representation of segmental duplications. We note similar proportions of bases in segmental duplications on chromosomal scaffolds (4,46% in Pan_tro_3.0 vs. 5,56% in GRCh38), however, we note an elevated genome wide rate of bases in duplications when including unplaced and unlocalized scaffolds, suggesting that our assembly includes false-positive paralogous regions within them (see supplementary Table 1).

By this means, we hope to clarify the questions of the reviewer: Our previous statements about segmental duplications being 'well resolved' referred to the comparable number of bases in segmental duplications between the Chimp and the Human assembly.

2. Lines 229 to 233. The finding is called "most striking" but it is accompanied by weak interpretation ("majority of … putative"). A little more investigation would probably support a strong claim of improvement. To estimate the veracity of the old frameshifts, clarify in what sense both assemblies are "mainly" based on data from the same individual, and measure if any frameshifted genes relied on reads from another individual. To estimate veracity of the new assemblies of these genes, rule out allelism in spanning reads, count framehshifts relative to human present in both assemblies, and count frameshifts exclusively in the new assembly. Presumably these counts are low.

We have now toned down this claim. We clarify to what extend the assemblies are based on the genomes of the same individual, and what proportions are derived from a different in Pan_tro_2.1.4 . We furthermore clarify, that these frameshifts don't necessarily constitute fixed changes, but might also be due to allelic variation. Furthermore, we have included the number of genes with predicted frameshifts only in Pan_tro_3, but not in Pan_tro_2.1.4

3. Line 125 "17 scaffold errors". Extrapolate the overall structural error rate using the number of bases spanned by fosmids. Extrapolate the likely number of remaining structural errors. Of errors fixed, where they attributable to the contig or the scaffold process specifically? Were the errors near particularly repetitive sequence?

Out of 671,716 fosmids with available end sequences for both ends from the CHORI-1251 library, 545,788 (81%) mapped with both ends and high quality (mismapping rate < 0.00001,

MQ<=60) onto the same scaffold. Out of these, we find 539,315 (99%) to map with both ends in concordant orientation, and 6,473 (1%) with both ends in the discordant orientation. Cumulatively, these concordant mappings cover 2.7 Gbps (85%) of the whole assembly length. As PBJelly changes the naming scheme of the sequences after filling gaps, we could not deduce at which stage the structural errors we fixed where introduced.

4. Line 129 and 232 “500,000 SNPs … frameshift”. Explanation, investigation, or speculation would help. What caused SNPs in contigs relative to the Illumina reads used to create the base assembly? What caused frameshifts in the prior assembly?

We now try to clarify by speculating that most of these corrected errors are due to regions where PacBio data was incorporated into the assembly (line 129). Given the relatively low coverage of PacBio data, we did not apply self correction on the PacBio reads, but rather corrected the assembly after filling in gaps. This leads to an elevated rate of residual errors in regions derived from PacBio data. We hypothesize that comparatively few of these corrected errors lay within regions derived from Illumina data. Given the constant change of coordinate system between the assemblies (with each incorporated platform) it is not straightforward to know which region in the assembly is finally derived from what platform.
We clarify that frameshifts with respect to Pantro2.1.4 are either because of allelic variation or sequencing errors in the Sanger data used to assemble Pantro2.1.4.

5. Line 65 “SMRTcells … synthetic long reads”. The manuscript does not address issues of integrating these technologies. Did either require error correction? Was there ever any overlap or disagreement between these two types of long reads? Was either more helpful than the other?

We had included an analysis of gap-filling performance and repeat resolution for PacBio and TruSeq SLR in the supplementary section S2, where we compare how well gaps in Pantro-2.1.4 are resolved using only either technology, as well as a combination of both. We did not incorporate the Truseq SLR data into the assembly based on the observed high rate of repeat collapse (see supplementary sections S2, supplementary Figures S2 and S3). Indeed, we see that many common high identity repeats are under-represented in sequencing data derived from this platform. We did not pre-correct the PacBio data, but rather run a post-assembly error correction, as described in the manuscript.

6. Line 223 “paralogous coding duplications are better represented”. What was the read coverage of these regions? Are these duplications specific to the chimp lineage or ancestral to primates? How is a paralogous coding duplication different from other kinds?

We now refrain to claim that, paralogous coding duplications are better represented’ as although we observe a shift to higher read depths in these regions, some of the newly added paralogs do no validate by excessive read depth.
7. Line 145 “bringing its continuity to the range”. The X chromosome N50 (422K) is actually larger than the average (385K). If the old assembly had smaller contigs at X due to half coverage of the X chromosome, then why isn’t that factor at play in the new assembly?

We believe this to be the case mainly due to two reasons: First, following a back of the envelope

calculation with the Lander-Waterman equation, there are about 5% of bases without a single read when sampling them at a 3X coverage. This relationship is non-linear with respect to coverage, and the number of unsampled bases drops to essentially 0 at a 30X coverage. Second, because of the initially poor assembly quality on the X, many BACs have been finished and integrated into the assembly. These BACs were also used for the final AGP creation of our assembly, boosting contiguity on this chromosome.

8. Line 126. Were SNPs concentrated in the gap fill regions? In the gaps filled with low-coverage long reads?

We speculate that this is most likely the case. However, given the constant change in coordinate system during the assembly, and also during the correction process itself, it is not straigthforward for us to keep track of the origin of each genomic region within the assembly.

9. Line 74 “finished BAC”. The BACs never get mentioned again. Do the old and new assemblies agree with the BACs?

We now clarify that these BACs were integrated into Pan_tro_2.1.4 as well as the finished version of Pan_tro_3. Thus, by definition, the final assemblies agree with the BACs

10. Line 76 “unprecedented”. Is there any need to make this controversial claim?

We have rephrased the sentence to tone down the claim.

11. Lines 89, 98, 128 “base assembly”. The DISCOVAR assembly is referenced by several names, some of which I confused with 2.1.4. Assign it a name or number?

The DISCOVAR base assembly is now consistently refered to as ‚DISCOVAR base assembly’

12. Line 92 should clarify this is a scaffold N50.

We clarified this regards the scaffold N50.

13. Line 95 “remaining gap structure required us to”. What is a gap structure? In what way was a response required?

We have rephrased this sentence to make it clearer.
