# Supplementary material for: A 3-way hybrid approach to generate a new high-quality chimpanzee reference genome (Pan_tro_3.0)
Source: Gigascience. 2017 Oct 30;6(11):1–6. doi: 10.1093/gigascience/gix098 (PMC5714192; doi:10.1093/gigascience/gix098)
Supplement: Reviewer-1-Report-(Original-Submission).pdf [file gix098_reviewer-1-report-(original-submission).pdf]

Reviewer Report

Title: A 3-way hybrid approach to generate a new high quality chimpanzee reference genome (Pan_tro_3.0)

Version: Original Submission **Date:** 12/20/2016

Reviewer name: Josh Burton

Reviewer Comments to Author:

The authors present several large new datasets of chimpanzee genome sequencing data, and they combine these datasets into a novel, high-quality genome assembly of Pan troglodytes. As the authors state, this is a valuable addition to the set of available genome sequence resources and a vast improvement in genome quality over the existing Pan troglodytes assembly. The manuscript needs some editing and cleaning up, but overall I believe it represents a significant contribution to the field and should eventually be published.In the "Data description" section, the paper gives an overview of the datasets the authors used. This section would benefit from a clear introduction and description of the sequencing strategies they employed to process these datasets. I suggest that a new figure, in the form of a simple flowchart, could be a helpful visual aid: it would describe the assembly methods that were used to combine the various types of sequencing libraries, and it would illustrate the process of creating the 3-way hybrid intermediary assembly as well as the final (3.0) assembly. Additionally, the "Data description" section is mostly devoid of citations. More citations should be added in order to give proper attribution to the developers of the assembly methods, and to enable the reader to seek more information.The authors discuss the sequence content they have added to the chimpanzee genome. It's interesting to see the length distribution of the gaps they have filled (Figure 1C), and I would be curious to see comparative length distributions for gaps they failed to fill, or for gaps they added. The detail on the repeat resolution is also fascinating. I think the authors sell themselves short by noting that the repeat fraction of the assembly increases from 50.9% to 52.2%: given that they only increase the assembly sequence length by ~8%, this actually shows that most of the sequence they've added is repeat sequence, which is a useful indicator of the new assembly's added value. Similarly, Figure 1D, which shows the quantities of added repeat sequence for various repeat types, would be stronger if it also showed the quantities of already-existing repeat sequence for each type.The authors compare the new (3.0) genome assembly to the existing (2.1.4) assembly. They observe a 99.9% overall sequence similarity and note that the 0.1% differences could be explained by SNPs; it would be interesting to see a deeper analysis of these SNPs, although this may be outside the scope of the manuscript. Also, in the section "Gene annotation", they note a large number of genes with frameshift mutations between the 2.1.4 assembly and the human genome assembly. This is striking, but a fully fair comparison would also mention the number of genes that also contain frameshift mutations (perhaps newly added frameshift mutations) in the 3.0 assembly.The conclusion is strong, but it would be stronger with some additional context that describes the achievement in this manuscript. The genome assembly is higher-quality. But is it also more efficient, or more economic? Have the authors innovated any new genome assembly methods? Have they demonstrated a technique that could be easily applied to other genome

assemblies?Minor errors:Section "Assembly generation": "These reads are derived from a 400 bps library, resulting in pairs that overlap over a ~50 bps region". If a 400-bp fragment is sequenced to 250 bp from both ends, wouldn't that result in an overlap of ~100 bp rather than ~50 bp?Section "Assembly generation": "we observed superior connectivity". The word "connectivity" is unclear in this context; it might be better to simply repeat "contiguity".Section "Repeat resolution": "We furthermore added 38.2 Mbp of LINE to the assembly, corresponding to over 44,791 additional copies of L1 elements." First of all, this should say "LINEs" rather than "LINE". Secondly, these numbers do not add up. A typical L1 element is 6 Kbp in length; thus, 44,791 copies of L1 elements should necessarily occupy over 260 Mbp of sequence.Section "Resolution of segmental duplications": This section should contain more citations, especially for the WGAC and WSSD methods, which are named but not described at all.

Level of Interest

Please indicate how interesting you found the manuscript: An article whose findings are important to those with closely related research interests

Quality of Written English

Please indicate the quality of language in the manuscript: Not suitable for publication unless extensively edited

Declaration of Competing Interests

Please complete a declaration of competing interests, considering the following questions:

- Have you in the past five years received reimbursements, fees, funding, or salary from an organisation that may in any way gain or lose financially from the publication of this manuscript, either now or in the future?
- Do you hold any stocks or shares in an organisation that may in any way gain or lose financially from the publication of this manuscript, either now or in the future?
- Do you hold or are you currently applying for any patents relating to the content of the manuscript?
- Have you received reimbursements, fees, funding, or salary from an organization that holds or has applied for patents relating to the content of the manuscript?
- Do you have any other financial competing interests?
- Do you have any non-financial competing interests in relation to this paper?

If you can answer no to all of the above, write 'I declare that I have no competing interests' below. If your reply is yes to any, please give details below.

I am a shareholder in Phase Genomics LLC.

I agree to the open peer review policy of the journal. I understand that my name will be included on my report to the authors and, if the manuscript is accepted for publication, my named report including any attachments I upload will be posted on the website along with the authors' responses. I agree for my report to be made available under an Open Access Creative Commons CC-BY license (http://creativecommons.org/licenses/by/4.0/). I understand that any comments which I do not wish to be included in my named report can be included as confidential comments to the editors, which will not be published.

I agree to the open peer review policy of the journal
