# Supplementary material for: A 3-way hybrid approach to generate a new high-quality chimpanzee reference genome (Pan_tro_3.0)
Source: Gigascience. 2017 Oct 30;6(11):1–6. doi: 10.1093/gigascience/gix098 (PMC5714192; doi:10.1093/gigascience/gix098)
Supplement: Reviewer-1-Report-(Revision-1).pdf [file gix098_reviewer-1-report-(revision-1).pdf]

**Reviewer Report**

**Title:** A 3-way hybrid approach to generate a new high quality chimpanzee reference genome (Pan_tro_3.0)

**Version:** Revision 1 **Date:** 6/13/2017

**Reviewer name:** Josh Burton

**Reviewer Comments to Author:**

Thank you very much for your response! You have addressed all of my concerns thoroughly and appropriately, and I believe this article is now ready for publication in its current form.

**Level of Interest**

Please indicate how interesting you found the manuscript: An article whose findings are important to those with closely related research interests

**Quality of Written English**

Please indicate the quality of language in the manuscript: Acceptable

**Declaration of Competing Interests**

Please complete a declaration of competing interests, considering the following questions:

- Have you in the past five years received reimbursements, fees, funding, or salary from an organisation that may in any way gain or lose financially from the publication of this manuscript, either now or in the future?
- Do you hold any stocks or shares in an organisation that may in any way gain or lose financially from the publication of this manuscript, either now or in the future?
- Do you hold or are you currently applying for any patents relating to the content of the manuscript?
- Have you received reimbursements, fees, funding, or salary from an organization that holds or has applied for patents relating to the content of the manuscript?
- Do you have any other financial competing interests?
- Do you have any non-financial competing interests in relation to this paper?

If you can answer no to all of the above, write 'I declare that I have no competing interests' below. If your reply is yes to any, please give details below.

I declare that I have no competing interests

I agree to the open peer review policy of the journal. I understand that my name will be included on my report to the authors and, if the manuscript is accepted for publication, my named report including any attachments I upload will be posted on the website along with the authors' responses. I agree for my report to be made available under an Open Access Creative Commons CC-BY license (http://creativecommons.org/licenses/by/4.0/). I understand that any comments which I do not wish to be included in my named report can be included as confidential comments to the editors, which will not be published.

I agree to the open peer review policy of the journal
