# Supplementary material for: A 3-way hybrid approach to generate a new high-quality chimpanzee reference genome (Pan_tro_3.0)
Source: Gigascience. 2017 Oct 30;6(11):1–6. doi: 10.1093/gigascience/gix098 (PMC5714192; doi:10.1093/gigascience/gix098)
Supplement: Reviewer-2-Report-(Original-Submission).pdf [file gix098_reviewer-2-report-(original-submission).pdf]

Reviewer Report

Title: A 3-way hybrid approach to generate a new high quality chimpanzee reference genome (Pan_tro_3.0)

Version: Original Submission **Date:** 3/8/2017

Reviewer name: Taras Oleksyk

Reviewer Comments to Author:

The genome of a chimpanzee is an important asset for the study of human evolution, and an high quality reference assembly is ling overdue and this assembly is long overdue. The authors were able to significantly improve on the previous assemblies. I have no problem with the 3-way hybrid approach they have taken. The paper is written very clearly, and is fully appropriate for the data note format. My recommendation is that this manuscript is accepted without reservation.

Level of Interest

Please indicate how interesting you found the manuscript: An article of importance in its field

Quality of Written English

Please indicate the quality of language in the manuscript: Acceptable

Declaration of Competing Interests

Please complete a declaration of competing interests, considering the following questions:

- Have you in the past five years received reimbursements, fees, funding, or salary from an organisation that may in any way gain or lose financially from the publication of this manuscript, either now or in the future?
- Do you hold any stocks or shares in an organisation that may in any way gain or lose financially from the publication of this manuscript, either now or in the future?
- Do you hold or are you currently applying for any patents relating to the content of the manuscript?
- Have you received reimbursements, fees, funding, or salary from an organization that holds or has applied for patents relating to the content of the manuscript?
- Do you have any other financial competing interests?
- Do you have any non-financial competing interests in relation to this paper?

If you can answer no to all of the above, write 'I declare that I have no competing interests' below. If your reply is yes to any, please give details below.

I declare that I have no competing interests

I agree to the open peer review policy of the journal. I understand that my name will be included on my report to the authors and, if the manuscript is accepted for publication, my named report including any attachments I upload will be posted on the website along with the authors' responses. I agree for my report to be made available under an Open Access Creative Commons CC-BY license (http://creativecommons.org/licenses/by/4.0/). I understand that any comments which I do not wish to be included in my named report can be included as confidential comments to the editors, which will not be published.

I agree to the open peer review policy of the journal
